# GhHUB2, a ubiquitin ligase, is involved in cotton fiber development via the ubiquitin–26S proteasome pathway

**DOI:** 10.1093/jxb/ery269

**Published:** 2018-07-20

**Authors:** Hao Feng, Xin Li, Hong Chen, Jie Deng, Chaojun Zhang, Ji Liu, Tao Wang, Xueyan Zhang, Jiangli Dong

**Affiliations:** 1 State Key Laboratory of Agrobiotechnology, College of Biological Sciences, China Agricultural University, Beijing, China; 2 State Key Laboratory of Cotton Biology, Institute of Cotton Research, Chinese Academy of Agricultural Sciences, Anyang, China

**Keywords:** Cotton fiber, *Gossypium hirsutum*, fiber elongation, HUB2, secondary cell wall (SCW), ubiquitin ligase, ubiquitin-26S proteasome pathway

## Abstract

Cotton fibers, which are extremely elongated single cells of epidermal seed trichomes and have highly thickened cell walls, constitute the most important natural textile material worldwide. However, the regulation of fiber development is not well understood. Here, we report that GhHUB2, a functional homolog of AtHUB2, controls fiber elongation and secondary cell wall (SCW) deposition. *GhHUB2* is ubiquitously expressed, including within fibers. Overexpression of *GhHUB2* in cotton increased fiber length and SCW thickness, while RNAi knockdown of *GhHUB2* resulted in shortened fibers and thinner cell walls. We found that GhHUB2 interacted with GhKNL1, a transcriptional repressor predominantly expressed in developing fibers, and that GhHUB2 ubiquitinated and degraded GhKNL1 via the ubiquitin-26S proteasome pathway. GhHUB2 negatively regulated GhKNL1 protein levels and lead to the disinhibition of genes such as *GhXTH1*, *Gh1,3-β-G*, *GhCesA4*, *GhAGP4*, *GhCTL1*, and *GhCOBL4*, thus promoting fiber elongation and enhancing SCW biosynthesis. We found that *GhREV-08*, a transcription factor that participates in SCW deposition and auxin signaling pathway, was a direct target of GhKNL1. In conclusion, our study uncovers a novel function of HUB2 in plants in addition to its monoubiquitination of H2B. Moreover, we provide evidence for control of the fiber development by the ubiquitin–26S proteasome pathway.

## Teaser

GhHUB2, a ubiquitin ligase, is involved in cotton fiber elongation and secondary cell wall deposition by degrading GhKNL1 via the ubiquitin–26S proteasome pathway, and thus demonstrates a novel function of HUB2 in plants.

## Introduction

Protein ubiquitination occurs in nearly every step of plant development (Smalle and Vierstra, 2004; Shu and Yang, 2017). The process of ubiquitination usually involves three enzymatic steps that are catalysed by ubiquitin-activating enzyme (E1), ubiquitin-conjugating enzyme (E2), and ubiquitin-protein isopeptide ligase (E3) (Deshaies and Joazeiro, 2009). Substrates can be monoubiquitinated or polyubiquitinated (Komander and Rape, 2012). Ubiquitin Lys48-linked polyubiquitination is usually coupled with protein degradation by the 26S proteasome. The ubiquitin–proteasome pathway has been well studied with respect to the regulation of plant developmental processes and responses to abiotic and biotic stresses (Nelson and Millar, 2015; Shu and Yang, 2017). However, the role of the ubiquitin–proteasome system (UPS) in controlling fiber development has not been characterized. Histone H2B monoubiquitination (H2Bub1) in plants can trigger seed dormancy, leaf and root growth, flowering, salt-stress tolerance, and defense responses (Fleury *et al.*, 2007; Liu *et al.*, 2007; Cao *et al.*, 2008, 2015; Hu *et al.*, 2014; Zou *et al.*, 2014; Zhou *et al.*, 2017). H2B can be ubiquitinated by the E3 ligases HUB1/HUB2 and E2 UBC1, UBC2, and UBC3. The role of HUB1 and HUB2 in triggering H2Bub1 has been studied in depth. Interestingly, studies have recently demonstrated that, in addition to H2Bub1, BRE1 and RNF20, which are homologs of HUB1 and HUB2 in yeast and mammalians, respectively, play roles in the regulation of polyubiquitination (Liu *et al.*, 2009; Lee *et al.*, 2014; Ren *et al.*, 2014; Silva *et al.*, 2015). It remains to be determined whether HUB1 and HUB2 function in plants in addition to H2Bub1, and in what processes they might participate.

Cotton (*Gossypium hirsutum*) fiber is the most important natural and renewable resource used in the textile industry (Wilkins *et al.*, 2000). Fibers are highly elongated and thickened single cells derived from epidermal cells on the outer integuments of the ovules (Basra and Malik, 1984). The development of cotton fibers can be divided into four distinct but overlapping stages: initiation, elongation, secondary cell wall (SCW) deposition, and maturation (Graves and Stewart, 1988). Recent studies have introduced an additional transition stage comprising the late-elongation stage and the early-SCW deposition stage (Hernandez-Gomez *et al.*, 2015; Li *et al.*, 2016). The SCW constitutes the major part of the mature fiber and determines its finesses and strength. The SCW of fibers is composed of nearly pure cellulose (Kim and Triplett, 2001). However, recent studies have demonstrated the existence of lignin/lignin-like phenolics in cotton fiber and have suggested that these correlate with fiber quality (Fan *et al.*, 2009; Han *et al.*, 2013).

Substantial progress has been made in identifying the regulators involved in fiber elongation and SCW formation (Ruan *et al.*, 2003; Li *et al.*, 2005, 2016; Han *et al.*, 2013; Xu *et al.*, 2013; Shan *et al.*, 2014; Lv *et al.*, 2015; Zhang *et al.*, 2018). A recent study examined the SCWs from different cotton tissues and identified several genes specially expressed in fibers, including two *AtKNAT7* homologs that were expressed during the fiber transition and SCW deposition stages (MacMillan *et al.*, 2017). Another study identified *GhKNL1*, a homolog of *AtKNAT7*, as a negative regulator of fiber development (Gong *et al.*, 2014). Overexpression of *GhKNL1* reduces the expression of *GhXTH1*, *Gh1,3-β-G*, the cell wall protein *GhAGP4*, and SCW biosynthesis genes such as *GhCesA4*, thus resulting in decreased fiber length and thinner SCWs. However, the mechanism that triggers GhKNL1 activity has not yet been elucidated.

In this study, we found that, compared with the wild-type, overexpression of *GhHUB2* significantly increased fiber length and SCW thickness. Discovery of this phenotype prompted us to examine the underlying molecular mechanisms. We determined that GhHUB2 interacts with the transcriptional repressor GhKNL1, which is predominantly expressed in developing fibers. GhHUB2 ubiquitinates GhKNL1 and directs its degradation through the ubiquitin–26S proteasome-dependent pathway. Our study thus reveals a novel function of HUB2 in plants.

## Materials and methods

### Plant material

The wild-type cotton (*Gossypium hirsutum*) variety CCRI24 was provided by the Institute of Cotton Research, Chinese Academy of Agricultural Sciences (CAAS). Wild-type and *GhHUB2* transgenic plants (T3 generation) were grown in the field at the experimental station of the Institute of Cotton Research, CAAS, and field management was performed according to standard local practices. Seeds were sown in rows that were 5 m in length with 25 plants, and the rows were spaced 0.8 m apart. Flowering time was recorded as days from the time of emergence. At least 30 plants of each line examined were used in the analyses. Cotton ovules were harvested at 0, 6, 10, 15, 20, and 30 d post anthesis (DPA). Fibers were scraped from ovules in liquid nitrogen. All materials were stored at –80 °C until use. The Arabidopsis *hub2-2* mutant (SALK_071289) was obtained from the Arabidopsis Biological Resource Center (https://abrc.osu.edu/). For the mutant complementation assays, 2 × 35S promoter-driven *GhHUB2* was transformed into *hub2-2* mutant plants using the floral dip method (Clough and Bent, 1998); the T3 generation was used for phenotypic analysis. Arabidopsis plants were grown at 22 °C and 65% relative humidity under a 16-h light photoperiod (70–100 μmol m^−2^ s^−1^). To determine flowering times, rosette leaf numbers were counted at bolting. Only lines that contained at least 32 plants were used for measurements.

### Plasmid construction and cotton transformation

For the construction of the *GhHUB2*-overexpression vector, the coding sequence (CDS) of *GhHUB2* was fused to a FLAG tag and cloned into the pMDC32 vector downstream of the 2 × 35S promoter (Curtis and Grossniklaus, 2003). To construct the *GhHUB2*-knockdown vector, a 272-bp region from the 3′ end of the *GhHUB2* CDS was chosen for RNAi. This fragment was recombined into pOSB209 to generate an RNAi cassette (Ma *et al.*, 2011), which was then cloned into a modified pBI121 vector. Transgenic cotton lines were generated by *Agrobacterium*-mediated transformation in accordance with previously described procedures (Zhang *et al.*, 2011*a*).

For the expression of His-MBP-GhHUB2, the CDS of *GhHUB2* was cloned into a modified pET-30a vector (Novagen/Merck) harboring maltose-binding protein (MBP). For the construction of the GST-GhKNL1 and His-GhKNL1 expression vectors, the CDS of *GhKNL1* was cloned into pGEX4T-1 (Pharmacia) and pET-30a, respectively.

### Phylogenetic analysis

DNA and protein sequences were analysed using the DNAMAN software (Lynnon Biosoft). The protein sequences were aligned using ClustalW (Thompson *et al.*, 1994) and an unrooted phylogenetic tree was constructed using the neighbor-joining method with the MEGA software version 5.1 (Tamura *et al.*, 2011). A bootstrap analysis with 1000 replicates was used to assess the consistency. Conserved protein domains were identified using SMART (Letunic *et al.*, 2015).

### Subcellular localization

The CDS of *GhHUB2* was cloned into the pNGFP (green fluorescent protein) vector to generate a GFP-GhHUB2 fusion construct. The CDS of *GhKNL1* was cloned into the pSAT6-RFP (red fluorescent protein) vector to generate a GhKNL1-RFP fusion construct. Both vectors contain 2 × 35S promoter. The plasmids were then transformed into Arabidopsis protoplasts using the polyethylene glycol transformation method (Bracha-Drori *et al.*, 2004; Walter *et al.*, 2004). GFP and RFP fluorescence were detected using confocal laser-scanning microscopy (Olympus FluoView FV1000 confocal fluorescence microscope). The intensity correlation analysis was performed as previously described (Duan *et al.*, 2017).

### RNA extraction and expression analysis

Total RNA was extracted from plant tissues using a RNAprep Pure Plant Kit (TIANGEN) in accordance with the manufacturer’s instructions. cDNA was synthesized using 2 µg of total RNA with Moloney murine leukemia virus reverse transcriptase, in accordance with the manufacturer’s instructions (Promega). Quantitative real-time PCR (qRT-PCR) was performed with a CFX-96 real-time system (Bio-Rad) using SYBR Green PCR MasterMix (TaKaRa).

### Yeast two-hybrid assays

The full-length CDS of *GhHUB2* was cloned into pDEST32 as bait to screen the cotton cDNA library using a ProQuest Two-Hybrid System (ThermoFisher Scientific) in accordance with the manufacturer’s instructions. To test specific interactions between GhHUB2 and GhKNL1, *GhHUB2* was cloned into pGADT7 as a prey vector, and *GhKNL1* or truncated *GhKNL1* was cloned into pGBKT7 as a bait vector. The bait and prey vectors were co-transformed into yeast strain AH109 and tested for interaction on SD medium.

### Yeast one-hybrid assays

Yeast one-hybrid assays were performed using a Matchmaker One-Hybrid Library Construction and Screening Kit (Clontech) in accordance with the manufacturer’s instructions. In brief, a 3× promoter fragment of *GhREV-08* was cloned into the pAbAibait vector, which was subsequently introduced into the yeast strain Y1H GOLD. Positive clones were cultured on SD/–Ura medium. The CDS of *GhKNL1* was cloned into the pGADT7 vector, which was subsequently transformed into yeast strains containing the pAbAi bait vector; these yeast strains were cultured on SD/–Leu medium. Positive clones were diluted and spotted on SD/–Leu medium containing 600 ng ml^–1^ AbA (Sigma-Aldrich).

### Detection of GhKNL1 abundance in cotton fibers

Cotton fibers at 6, 10, 15, and 20 DPA were ground to a fine powder in liquid nitrogen. Nuclear proteins were then extracted as previously described (Du *et al.*, 2015; Duan *et al.*, 2017). The extracted proteins were quantified using a Coomassie (Bradford) protein assay kit (Pierce). For the detection of GhKNL1 abundance, equal amounts of nuclear proteins were separated by SDS-PAGE and detected by immunoblotting by using anti-H3 and anti-GhKNL1 antibodies. The polyclonal antibody against GhKNL1 was prepared by BGI Tech. In brief, His-GhKNL1 protein purified from *E. coli* was used to immunize rabbits for the production of polyclonal antiserum. Antigen affinity-purified anti-GhKNL1 antibodies (1:2000) were used in immunoblots. Other commercial antibodies used in this study are listed in Supplementary Table S3at *JXB* online.

### Transient GUS activity assays

For the detection of the transcriptional repression activity of GhKNL1, the CDS of GhKNL1 was fused to GAL4BD to generate GAL4BD-GhKNL1, which was then cloned into pCAMBIA1302 under the control of the 35S promoter. Transient expression assays were performed as previously described (Tao *et al.*, 2013). For the transient analysis of GhKNL1 repressing the expression of GhREV-08, a 2000-bp upstream fragment of GhREV-08 was cloned into pCAMBIA1381 to drive the expression of the GUS (β-glucuronidase) reporter gene. The analysis was performed as previously described (Duan *et al.*, 2017).

### Firefly luciferase complementation assays

The CDS of *GhHUB2* and *GhSINA* were fused to the C-terminus of the pCAMBIA-cLUC (luciferase) vector, while *GhKNL1* and *GhHOX3* were fused to the N-terminus of the pCAMBIA-nLUC vector. Firefly luciferase complementation assays were conducted as previously described (Kong *et al.*, 2015).

### Co-immunoprecipitation

Co-immunoprecipitation (Co-IP) was conducted as previously described (Liu *et al.*, 2010; Shen *et al.*, 2016). In brief, MYC-GhKNL1-GFP and FLAG-GhHUB2 or FLAG-GFP were transiently co-expressed in tobacco leaves for 3 d. At 14 h before sample collection, 100 µM 26S proteasome inhibitor MG132 was infiltrated, after which a native buffer [50 mM Tris-MES (pH 8.0), 0.5 M sucrose, 1 mM MgCl_2_, 10 mM EDTA, 5 mM DTT and protease inhibitor cocktail (Roche)] was used to extract soluble proteins. Lysates were incubated with anti-FLAG-affinity M2 beads (Sigma-Aldrich) at 4 °C for 2 h. The beads were then washed three times with PBS. The immunoprecipitated proteins were then examined by immunoblotting.

### *In vitro* ubiquitination

His-MBP-GhHUB2, His-MBP, and GST-GhKNL1 proteins were purified from *E. coli* strain BL21 (DE3). *In vitro* ubiquitination assays were conducted as previously reported (Yang *et al.*, 2015).

### *In vivo* and semi-*in vivo* protein degradation assays

*In vivo* degradation assays were performed in accordance with previous reported methods (Yang *et al.*, 2015; Shen *et al.*, 2016). Semi-*in vivo* degradation assays were performed as previously described (Liu *et al.*, 2010; Shen *et al.*, 2016).

### Measurements of cellulose and lignin/lignin-like phenolics content in cotton fibers

The crystalline cellulose content was measured as described previously (Li *et al.*, 2013, 2016). Fiber staining with phloroglucinol-HCl and measurements of fiber lignin/lignin-like phenolics content were conducted as described by Han *et al.* (2013). Three replicates were measured, and each replicate contained 15 samples.

Autofluorescence of fiber lignin/lignin-like phenolics was observed via excitation at 488 nm in accordance with previous methods (Mast *et al.*, 2009). For the detection of cellulose, cotton fibers were stained with 0.005% Calcofluor white for 1 min and observed under UV light (405 nm) as described previously (Zhong *et al.*, 2006; Mendu *et al.*, 2011).

### EMSA

His-GhKNL1 protein was purified from *E. coli*. EMSA was conducted using biotin-labeled probes and a Lightshift Chemiluminescent EMSA kit (ThermoFisher) as described previously (Han *et al.*, 2013; Zhang *et al.*, 2016).

### Accession numbers

Sequence data can be found in GenBank/NCBI under the following accession numbers: *GhHUB2-A* (XM_016856414), *GhHUB2-D* (XM_016843047), *GhKNL1* (KC200250). Other accession numbers related to this study are listed in Supplementary Table S4.

## Results

### Cloning of *GhHUB2* from upland cotton

**Fig. 1.**
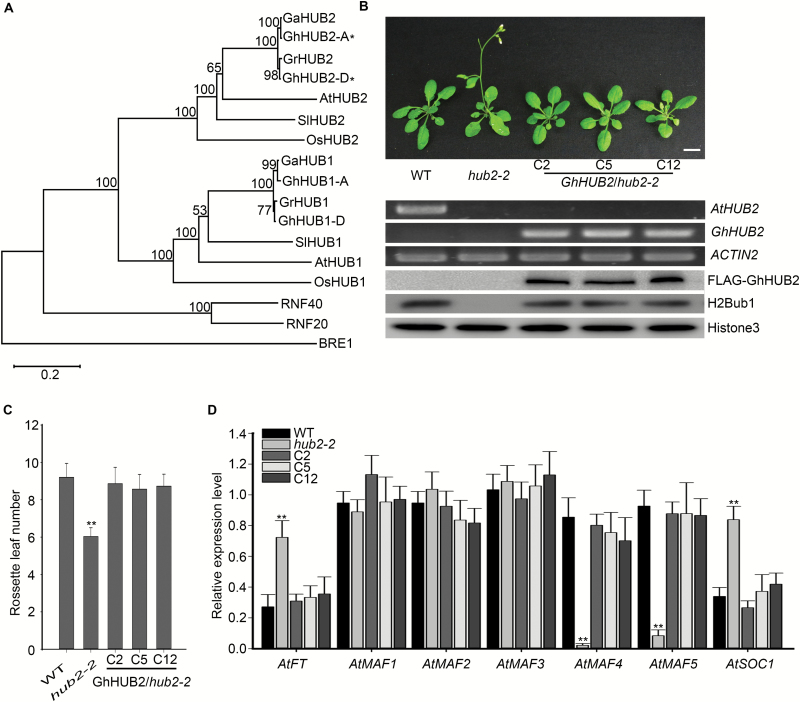
Phylogenetic analysis of GhHUB2 and complementation of the Arabidopsis *hub2* mutant by *GhHUB2*. (A) Phylogenetic analysis of GhHUB2 homologs. Gh, *Gossypium hirsutum*, Ga, *G. arboreum*, Gr, *G. raimondii*, At, *Arabidopsis thaliana*, Os, *Oryza sativa*, Sl, *Solanum lycopersicum*. GhHUB2-A and GhHUB2-D are indicated with asterisks. (B) Complementation of the Arabidopsis *hub2* mutant by ectopic expression of *GhHUB2*. The phenotypes of the Col-0 wild-type (WT), *hub2-2*, and *hub2-2* transformed with *GhHUB2* (C2, C5, and C12) are shown. The scale bar is 1 cm. RT-PCR was used to detect the expression of *AtHUB2* and *GhHUB2*; *ACTIN2* was used as an internal control. Immunoblotting using antibodies against FLAG and H2Bub1 in extracted nuclear proteins was used to determine abundance; Histone3 was used as a loading control. (C) The number of rosette leaves at bolting. Data are means (±SD), *n*≥32 (**P<0.01, Student’s *t*-test). (D) Expression of *FT*, *MAF*s, and *SOC1* in 10-d-old seedlings of the WT, *hub2-2* mutants, and *hub2-2* transformed with *GhHUB2*. *ACTIN2* was used as the internal control. Data are means (±SE) of triplicate experiments. Significant differences compared with the WT were determined using Student’s *t*-test; **P<0.01.

To investigate the function of HUB2 in upland cotton, we searched the sequenced *G. hirsutum* accession TM-1 genome (Zhang *et al.*, 2015) using the AtHUB2 protein sequence and identified two candidate homologs that we named GhHUB2-A and GhHUB2-D. We then phylogenetically analysed a selected set of HUB2 proteins from various species to explore their evolutionary relationships. The results showed that GhHUB2-A and GhHUB2-D both fell within the clade containing previously characterized HUB2s, including OsHUB2, SlHUB2, and AtHUB2 (Fig. 1A). A multiple sequence alignment indicated that GhHUB2-A and GhHUB2-D shared 98.4% amino acid sequence identity and 98.6% nucleotide identity, respectively (see Supplementary Fig. S1B). Both homologs shared nearly 60% sequence identity with that of AtHUB2. Conserved-domain search results indicated that GhHUB2s and AtHUB2 both contained a conserved C3HC4 RING finger domain in their C-terminal regions and several coiled coil domains along the whole protein (Supplementary Fig. S1A). Furthermore, *in silico* analysis revealed similar genomic architecture between the gene structures of *AtHUB2*, *GhHUB2-A*, and *GhHUB2-D*. Each gene comprised 19 exons and 18 introns. *GhHUB2-D* was chosen for further study and is henceforth referred to as *GhHUB2*.

To confirm that GhHUB2 is a homolog of AtHUB2, the CDS of *GhHUB2* was introduced into Arabidopsis *hub2-2* mutant plants (SALK_071289) under the control of 2 × 35S promoter. A total of 21 positive lines were identified by PCR and inbred T3 lines were used for analysis. The *hub2-2* mutant displays an early-flowering phenotype, but ectopic expression of *GhHUB2* in *hub2-2* mutant plants significantly delayed their flowering time, restoring it to nearly the same as that exhibited by the wild-type (Fig. 1B), a result that was also confirmed by statistical analysis (Fig. 1C). Previous studies have reported that the *hub2-2* mutant has lost H2Bub1 and exhibits both low expression of *MAF*s and high expression of *FT* and *SOC1* (Cao *et al.*, 2008). Our immunoblotting results showed that GhHUB2 rescued H2Bub1 in the *hub2-2* mutant (Fig. 1B), and expressing *GhHUB2* in the *hub2-2* mutants altered the expression levels of *MAF*s, *FT*, and *SOC1* (Fig. 1D). Taken together, our results demonstrated that GhHUB2 plays a role in H2Bub1 and is a functional homolog of AtHUB2 in upland cotton.

### Molecular characterization of *GhHUB2*

To study the expression pattern of *GhHUB2*, a 2-kb upstream DNA fragment of GhHUB2 was fused to a GUS reporter gene and introduced into Arabidopsis. A total of 25 positive lines were identified by PCR. Histochemical staining was performed on various organs of T3 plants. The results showed that *GhHUB2* was expressed ubiquitously in seedlings, leaves, roots, and flowers (Supplementary Fig. S2), and further analysis also showed moderate GUS activity in the trichomes of rosette leaves. Arabidopsis trichomes and cotton fiber may share a common regulatory mechanism and development process (Guan *et al.*, 2007). In addition, many genes preferentially expressed in fibers show trichome-specific expression patterns when heterogeneously expressed in Arabidopsis (Suo *et al.*, 2003; Wang *et al.*, 2004; Shangguan *et al.*, 2008; Li *et al.*, 2015). The expression of *GhHUB2* in trichomes suggested that it may function in fiber development.

**Fig. 2.**
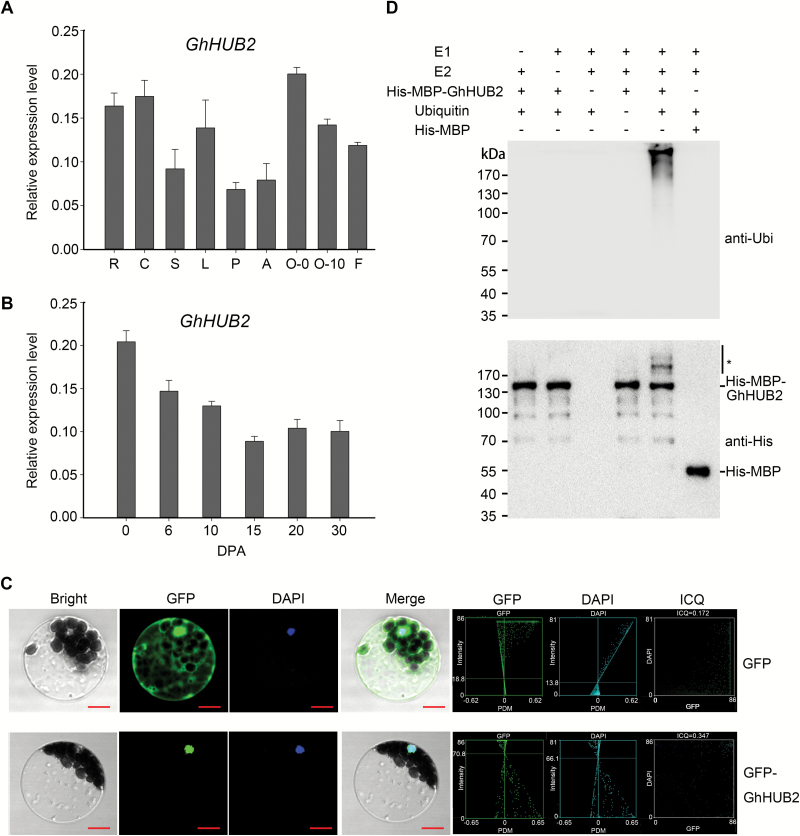
Molecular characterization of *GhHUB2*. (A) Expression of *GhHUB2* in roots (R), cotyledons (C), stems (S), leaves (L), petals (P), anthers (A), ovules at 0 d post-anthesis (DPA, O-0), ovules at 10 DPA (O-10), and fibers at 10 DPA (F), as analysed by qRT-PCR. *Histone3* was used as the internal control. Data are means (±SE) of triplicate experiments. (B) Expression of *GhHUB2* in developing cotton fibers between 0–30 DPA, as analysed by qRT-PCR. *Histone3* was used as the internal control. Data are means (±SE) of triplicate experiments. (C) Subcellular localization of GhHUB2 in Arabidopsis protoplasts. GhHUB2 was fused to the C-terminus of green fluorescent protein (GFP) and was expressed under the control of the 35S promoter. The GFP and DAPI (4′,6-diamidino-2-phenylindole) signals were observed at 488 nm and 358 nm excitation wavelengths, respectively. The intensity correlation quotient (ICQ) value of GFP-GhHUB2 against DAPI is 0.347: usually, an ICQ value between 0 and 0.5 represents dependent staining and indicates co-localization. Scale bars are 10 µm. (D) *In vitro* autoubiquitination assays of GhHUB2. His-MBP-GhHUB2 fusion proteins were used to test E3 ubiquitin ligase activity in the presence of E1, E2, and ubiquitin. His-MBP protein was used as a negative control. Ubiquitination of His-MBP-GhHUB2 was detected by antibodies against ubiquitin (anti-Ubi) and His (anti-His). * indicates ubiquitinated His-MBP-GhHUB2.

We also carried out quantitative real-time PCR (qRT-PCR) to analyse *GhHUB2* transcripts in various organs throughout cotton development. The result confirmed that *GhHUB2* was broadly expressed in all the organs examined, including cotyledons, roots, stems, leaves, flowers, and ovules (Fig. 2A). As expected, moderate expression was detected in developing fibers (20 DPA), and the qRT-PCR results i showed that *GhHUB2* was constitutively expressed throughout fiber development (Fig. 2B), indicating that it may participate in the regulation of fiber development.

To determine the subcellular localization, GhHUB2 was fused in-frame to the C-terminus of GFP and transformed into Arabidopsis mesophyll protoplasts. The results showed that GFP-GhHUB2 was localized to the nucleus (Fig. 2C). We tested the autoubiquitination activity of GhHUB2 *in vitro* and found that a polyubiquitination signal was detected by immunoblotting using anti-His and anti-Ubi antibodies when His-MBP-GhHUB2 was added to the reaction. However, no polyubiquitination signal was detected when E1, E2, or ubiquitin was absent or when the His-MBP-GhHUB2 was replaced with His-MBP protein (Fig. 2D). These results suggested that GhHUB2 is a functional E3 ubiquitin ligase.

### GhHUB2 controls cotton flowering time via H2Bub1

To gain insight into the biological role of GhHUB2 during cotton development, 17 *GhHUB2*-overexpression (-OE) and 15 RNAi-knockdown lines were generated using *Agrobacterium tumefaciens*-mediated transformation. Three *GhHUB2*-OE lines (402–13, 402–20, and 402–47) and three *GhHUB2*-knockdown lines (401–34, 401–52, and 401–57) were chosen for further analysis. T3 generations were obtained by successive inbreeding and the expression levels of *GhHUB2* in the transgenic lines were confirmed by qRT-PCR (Supplementary Fig. S3A, B). *GhHUB2* transgenic cotton showed no significant alteration in trichome development compared with the wild-type. We then examined whether ectopic expression of *GhHUB2* disrupted cotton flowering time. The results showed that overexpression of *GhHUB2* significantly delayed flowering compared with the wild-type (Supplementary Fig. S3C, D). Further analysis showed that overexpression of *GhHUB2* clearly increased H2Bub1 levels and decreased the expression of the homologs of *FT* and *SOC1* (Supplementary Fig. S3F, G). Contrasting results were observed in the *GhHUB2*-knockdown lines. These results demonstrated that GhHUB2 plays a conserved role in the regulation of flowering time via fine-tuning of H2Bub1.

### GhHUB2 is associated with fiber length and SCW thickness

**Fig. 3.**
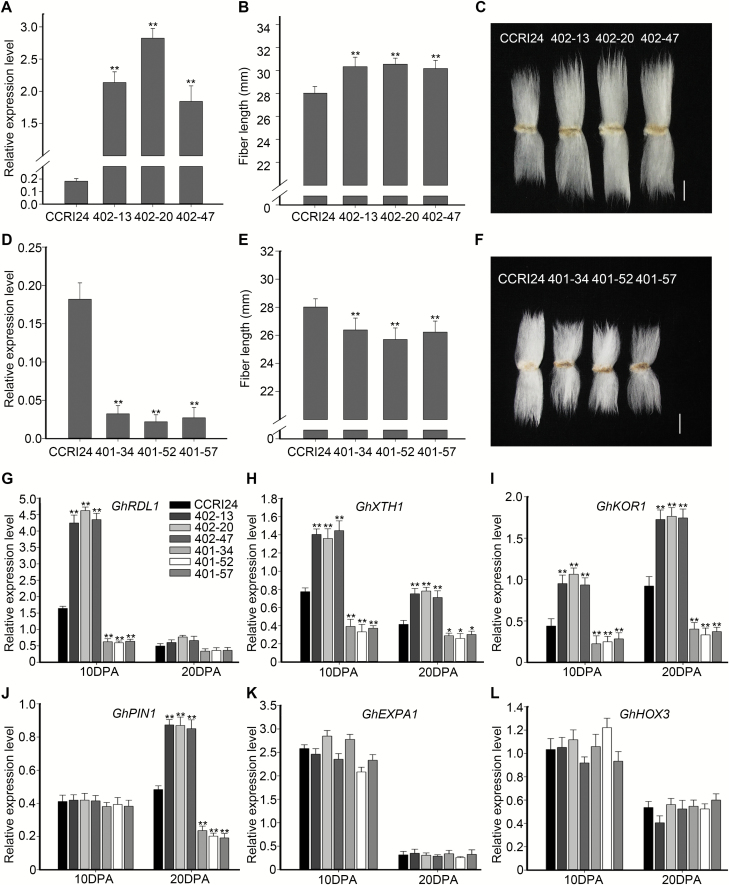
GhHUB2 affects cotton fiber length and the expression of genes involved in fiber elongation in transgenic cotton. (A, D) qRT-PCR analysis of the expression of *GhHUB2* in fibers of wild-type (WT) CCRI24 and the *GhHUB2*-OE (overexpression) (A) and *GhHUB2*-knockdown lines (D), at 10 d post-anthesis (DPA). *Histone3* was used as the internal control. Data are means (±SE) of triplicate experiments (**P<0.01, Student’s *t*-test). (B, E) Comparison of mature fiber lengths of the WT and the *GhHUB2*-OE (B) and *GhHUB2*-knockdown (E) lines. Data are means (±SE), *n*=30. Significant differences compared with the WT were determined using Student’s *t*-test; **P<0.01. (C, F) Phenotypes of fibers from wild-type CCRI24 and the *GhHUB2*-OE (C) and *GhHUB2*-knockdown (F) lines. Scale bars are 1 cm. (G–L) qRT-PCR analysis of genes involved in fiber elongation in fibers from transgenic lines and wild-type at 10 DPA and 20 DPA, showing the expression of *GhRDL1* (G), *GhXTH1* (H), *GhKOR1* (I), *GhPIN1* (J), *GhEXPA1* (K), and *GhHOX3* (L). *Histone3* was used as the internal control. Data are means (±SE) of triplicate experiments. Significant differences compared with the WT were determined using Student’s *t*-test; *P<0.05, **P<0.01.

As *GhHUB2* was expressed at different developmental stages of fibers, we investigated the effect of GhHUB2 on fiber properties in transgenic cotton plants from the T3 generation. qRT-PCR results confirmed that the expression of *GhHUB2* was elevated in *GhHUB2*-OE fibers and reduced in *GhHUB2*-knockdown fibers (Fig. 3A, D). Compared with that of wild-type, the mature fiber length of the *GhHUB2*-OE lines was increased by 7.49–8.99% (Fig. 3B, C). In contrast, the length of *GhHUB2*-knockdown fibers was much shorter than that of wild-type fibers, exhibiting a reduction of 5.85–8.24% (Fig. 3E, F). Using *in vitro* ovule culture, we confirmed the effect of GhHUB2 on fiber length (Supplementary Fig. S4). These results indicated that GhHUB2 is associated with fiber length.

We then examined the expression of genes reportedly responsible for fiber elongation. The results showed that the BURP-domain cell wall protein *GhRDL1*, the auxin transporter *GhPIN1*, and two glucanases (*GhXTH1* and *GhKOR1*) were up-regulated in *GhHUB2*-OE fibers and down-regulated in *GhHUB2*-knockdown fibers. However, the expressions of another cell wall protein, *GhEXPA1*, and the homeodomain transcription factor *GhHOX3* were comparable to that of the wild-type (Fig. 3G–L). These results suggested that the changes in transgenic fiber length may have been due to the altered expression of *GhRDL1*, *GhPIN1*, *GhXTH1*, and *GhKOR1*.

**Fig. 4.**
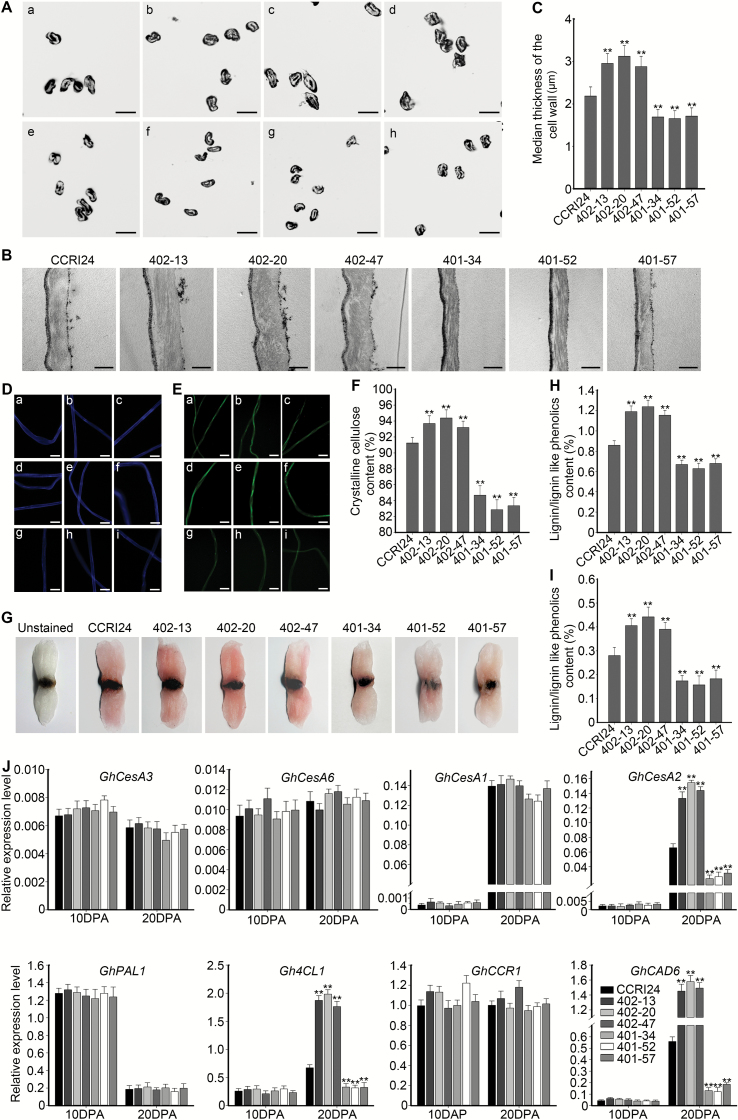
Analysis of fiber cell wall thickness, contents of cellulose and lignin/lignin-like phenolics, and fiber cell biosynthesis genes in transgenic cotton. (A) Cross-sections of mature fibers: (a, e) fibers from the wild-type (WT) CCRI24; (b–d) fibers from the *GhHUB2*-OE (overexpression) lines 402–13, 402–20, and 402–47; and (f–h) fibers from the *GhHUB2*-knockdown lines 401–34, 401–52, and 401–57. Scale bars are 20 µm. (B) TEM of WT and transgenic fibers at 30 d post-anthesis (DPA). Scale bars are 2 µm. (C) Median thickness of mature fiber cell walls in WT and transgenic lines. Data are means (±SE), *n*≥300. Significant differences compared with the WT were determined using Student’s *t*-test; **P<0.01. (D) Fluorescence imaging of mature fibers stained with Calcofluor white to detect cellulose: (a–c) fibers from the WT; (d–f) fibers from the *GhHUB2*-OE lines 402–13, 402–20, and 402–47; and (g–i) fibers from the *GhHUB2*-knockdown lines 401–34, 401–52, and 401–57. Scale bars are 20 µm. (E) Autofluorescence images of lignin/lignin-like phenolics of mature fibers: (a–c) fibers from the WT; (d–f) fibers from the *GhHUB2*-OE lines 402–13, 402–20, and 402–47; and (g–i) fibers from the *GhHUB2*-knockdown lines 401–34, 401–52, and 401–57. Scale bars are 20 µm. (F) Mature crystalline cellulose contents of WT and transgenic fibers. Data are means (±SE) of three experimental replicates, *n*=30. Significant differences compared with the WT were determined using Student’s *t*-test; **P<0.01. (G) Phloroglucinol-HCl staining of mature WT (CCRI24) and transgenic fibers. Representative images are shown, including the unstained WT. (H, I) Lignin/lignin-like phenolics content in mature fibers measured by Klason extraction (H) and thioglycolate analysis (I). Data are means (±SE) of three experimental replicates, *n*=30. Significant differences compared with the WT were determined using Student’s *t*-test; **P<0.01. (J) qRT-PCR analysis of genes involved in fiber cell wall biosynthesis in fibers from the WT (CCRI24) and transgenic lines at 10 DPA and 20 DPA. *Histone3* was used as the internal control. Data are means (±SE) of triplicate experiments. Significant differences compared with the WT were determined using Student’s *t*-test; **P<0.01.

Since the SCW constitutes the major component of the mature fiber, we examined the cell wall thickness of transgenic cotton lines compared with the wild-type. Cross-sections showed that overexpression of *GhHUB2* resulted in significantly thicker fiber walls (Fig. 4A). Compared with that of wild-type (2.19 µm), the median wall thicknesses of the *GhHUB2*-OE lines were significantly increased (2.95, 3.12, and 2.88 µm) (Fig. 4C). In contrast, cell wall thickness decreased in the fibers of the *GhHUB2*-knockdown lines (1.69, 1.66, and 1.71 µm) (Fig. 4A, C); this was further confirmed by TEM (Fig. 4B). Thus, our results suggested that GhHUB2 may be involved in fiber SCW deposition.

To further explore the role of GhHUB2 in fiber development, more detailed measurements of fiber qualities of the transgenic cotton lines (T4 generation) were made at the Center of Cotton Fiber Quality Inspection and Testing, Chinese Ministry of Agriculture (Anyang, Henan Province, China) (Supplementary Table S1). These confirmed that GhHUB2 was associated with altered fiber length. In addition, the micronaire value of *GhHUB2*-OE fibers clearly increased, which was consistent with the thickened SCW (this value provides an indication of the linear density and degree of cell-wall development). Moreover, the thickened SCW may also have contributed to elevated fiber strength. Taken together, the changes in fiber quality suggested that GhHUB2 participates in the regulation of fiber length and SCW deposition.

### GhHUB2 modulates the contents of cellulose and lignin/lignin-like phenolics in cotton fibers

To determine the reason for the thickened SCWs, we first examined the content of cellulose in fibers. Cellulose can be stained by Calcofluor white (Zhong *et al.*, 2006, 2007) and we found that the stained fibers of the *GhHUB2*-OE lines fluoresced brighter under UV stimulation than those of the wild-type; in contrast, the *GhHUB2*-knockdown fibers fluoresced more weakly (Fig. 4D). We then measured the crystalline cellulose contents in transgenic and wild-type cotton fibers. Compared with wild-type, elevated expression of *GhHUB2* significantly increased the crystalline cellulose content, by 2.13–3.44%; in contrast, the content in the *GhHUB2*-knockdown lines was reduced by 7.20–9.19% (Fig. 4F). These results indicated that the altered content of cellulose in the transgenic cotton fibers may have accounted for the observed changes in SCW thickness.

Previous studies have shown that lignin/lignin-like phenolics exist in cotton fibers and contribute to enhanced fiber strength (Al-Ghazi *et al.*, 2009; Fan *et al.*, 2009; Han *et al.*, 2013). To investigate whether the thickened SCW and increased fiber strength in cotton also resulted from altered lignin/lignin-like phenolics content, we analysed their deposition. Fibers were first stained with phloroglucinol-HCl in accordance with previous studies (Han *et al.*, 2013), and the color of the *GhHUB2*-OE lines was clearly darker red, indicating elevated contents of lignin/lignin-like phenolics (Fig. 4G). This result was confirmed by examining the lignin/lignin-like phenolics autofluorescence of fibers under UV stimulation. Relatively strong autofluorescence was observed in *GhHUB2*-OE fibers; in contrast, *GhHUB2*-knockdown fibers were stained a lighter color and showed weaker autofluorescence (Fig. 4E). Using the Klason method, we found that acid-insoluble lignin/lignin-like phenolics constituted 0.86% in the wild-type fibers and 1.19, 1.24, and 1.15% in the *GhHUB2*-OE lines (Fig. 4H). Using the thioglycolate method, we found that acid-soluble lignin/lignin-like phenolics also significantly increased in the *GhHUB2*-OE lines (Fig. 4I). Contrasting results were found in the *GhHUB2*-knockdown lines. Taken together, the results demonstrated that, in addition to cellulose, altered lignin/lignin-like phenolics content may also have contributed to the changes in the SCW and fiber strength of the transgenic lines.

To explore the molecular mechanism that triggered SCW thickening in transgenic fibers, we examined the expression of genes involved in the biosynthesis of cellulose and lignin/lignin-like phenolics in fibers at 10 DPA and 20 DPA. The expression of *GhCesA2*, a cellulose synthase A that participates in SCW biosynthesis, significantly increased in the *GhHUB2*-OE fibers (Fig. 4J). In contrast, its expression was reduced in the *GhHUB2*-knockdown lines. The expression of other SCW cellulose synthetase As, *GhCesA1* (Fig. 4J), *GhCesA7* and *GhCesA8* (Supplementary Fig. S5), and two primary cell wall cellulose synthetase As, *GhCesA3* and *GhCesA6* (Fig. 4J), remained unchanged. Among the four genes examined whose products catalyse lignin/lignin-like phenolics biosynthesis, we found that the expression of *Gh4CL1* and *GhCAD6* increased 2–3-fold in the *GhHUB2*-OE lines, but expression was suppressed to 20–50% of the wild-type value in the *GhHUB2*-knockdown fibers (Fig. 4J). The results indicated that the changes in fiber SCW deposition resulted from changes in the biosynthesis of both cellulose and lignin/lignin-like phenolics.

### GhHUB2 interacts with the transcription factor GhKNL1 that is preferentially expressed in fibers

**Fig. 5.**
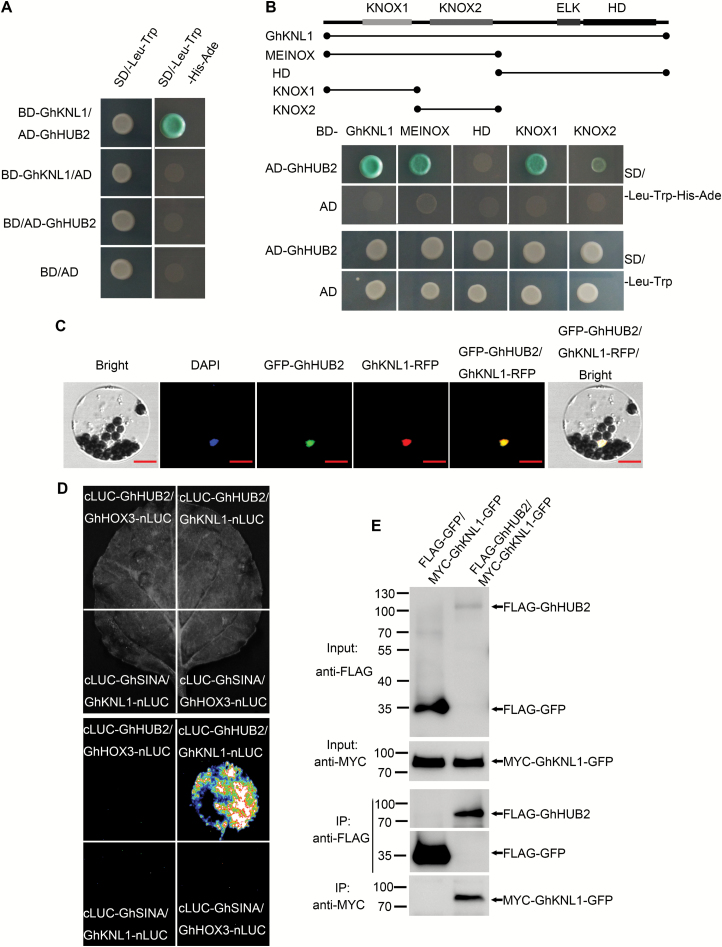
GhHUB2 interacts with GhKNL1 and co-localizes with GhKNL1 in the nucleus. (A) GhHUB2 interacts with full-length GhKNL1 in yeast. (B) KNOX1 and KNOX2 domains in GhKNL1 mediate the interaction of GhHUB2 and GhKNL1. (C) GhHUB2 and GhKNL1 co-localize in the nucleus. Scale bars are 10 µm. (D) GhHUB2 interacts with GhKNL1 in a split firefly luciferase complementation assay. GhHUB2 fused to the C-terminus of LUC (cLUC-GhHUB2) was co-expressed with a GhKNL1-fused N-terminus of LUC (GhKNL1-nLUC) in *Nicotiana benthamiana* leaves. Images were taken 3 d after infiltration. The RING domain E3 ligase GhSINA and the homeodomain transcription factor GhHOX3 were used as negative controls. (E) GhHUB2 interacts with GhKNL1 in a co-immunoprecipitation (Co-IP) assay. FLAG-GhHUB2 and MYC-GhKNL1-GFP were co-expressed in *N. benthamiana* leaves, and the 26S proteasome inhibitor MG132 (100 µM) was infiltrated 14 h before sample collection. Co-IP was carried out with anti-FLAG agarose from the total isolated proteins, and immunoblotting analysis was performed with anti-FLAG and anti-MYC antibodies. Full size images are shown in Supplementary Fig. S6.

To investigate the molecular mechanisms governing the triggering of fiber development by GhHUB2, we performed yeast two-hybrid assays using GhHUB2 as bait in order to screen for interacting proteins in a cDNA library encompassing all cotton developmental stages. Not surprisingly, we identified GhHUB1, GhUBC1, and GhH2B, which are involved with H2Bub1. Interestingly, seven independent clones encoding the homeodomain transcription factor GhKNL1 were also identified (Supplementary Table S2). We cloned the full-length GhKNL1 and verified its interaction with GhHUB2 in yeast cells (Fig. 5A). Further analysis showed that the KNOX domain (either KNOX1 or KNOX2) mediated the interaction between GhKNL1 and GhHUB2 (Fig. 5B). This interaction was confirmed by firefly luciferase complementation and Co-IP assays (Fig. 5D, E, Supplementary Fig. S6). When co-expressed with GFP-GhHUB2, GhKNL1-RFP as well as GFP-GhHUB2 co-localized to the nucleus (Fig. 5C). Taken together, these results suggested that GhHUB2 interacts with GhKNL1, and the interaction occurs in the nucleus.

### GhHUB2 ubiquitinates GhKNL1 and promotes its degradation

**Fig. 6.**
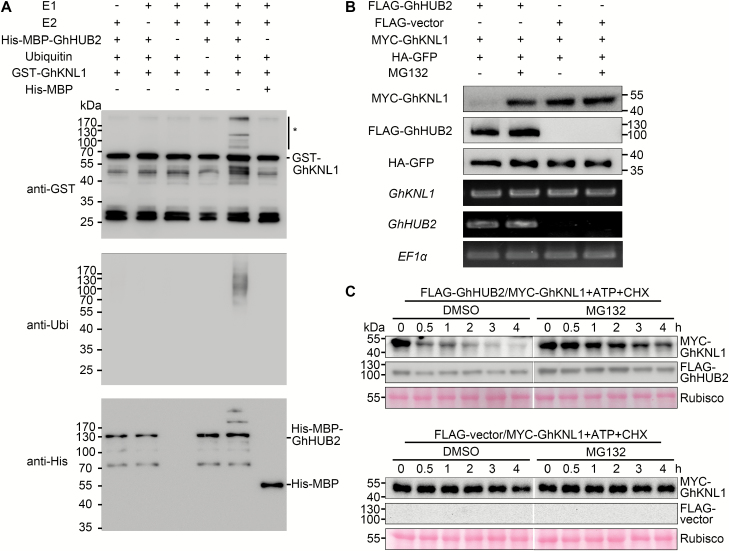
GhHUB2 ubiquitinates GhKNL1 and promotes the degradation of GhKNL1. (A) GhHUB2 ubiquitinates GhKNL1 *in vitro*. Ubiquitinated GST-GhKNL1 was detected by anti-GST and anti-Ubi antibodies. * indicates ubiquitinated GST-GhKNL1. (B) GhHUB2 promotes the degradation of GhKNL1 via the 26S proteasome *in vivo*. Immunoblotting analysis of protein extracts corresponding to agroinfiltrated *Nicotiana benthamiana* leaves with the indicated plasmids in the presence or absence of MG132. The abundance of MYC-GhKNL1 was detected using anti-MYC antibody and that of FLAG-GhHUB2 using anti-FLAG antibody. HA-GFP detection using anti-HA antibody served as a loading control. The mRNA expression levels of *FLAG-GhHUB2* (*GhHUB2*) and *MYC-GhKNL1* (*GhKNL1*) were analysed by RT-PCR; the mRNA expression level of *EF1α* was used as the internal control. (C) GhHUB2 promotes the degradation of GhKNL1 in a semi-*in vivo* protein degradation assay. FLAG-GhHUB2, MYC-GhKNL1, or FLAG vectors were individually expressed in *N. benthamiana* leaves for protein extraction. Extracts from MYC-GhKNL1 were mixed together with FLAG-GhHUB2 or FLAG vectors, after which the mixtures were treated with CHX and ATP in the presence of MG132 or DMSO for different times at 25 °C. At each time point, a portion of the mixture was removed and analysed by immunoblotting. The abundance of MYC-GhKNL1 was detected using anti-MYC antibody, and FLAG-GhHUB2 was detected using anti-FLAG antibody. Ponceau staining of Rubisco was used as a loading control.

Recent studies in yeast and mammalian cells have demonstrated new roles for BRE1 and its homolog RNF20 in the regulation of polyubiquitination (Liu *et al.*, 2009; Lee *et al.*, 2014; Ren *et al.*, 2014; Silva *et al.*, 2015). Our results demonstrated that GhHUB2 interacted with GhKNL1 *in vivo*. To examine whether the interaction resulted in the ubiquitination of GhKNL1, we performed *in vitro* ubiquitination assays using purified His-MBP-GhHUB2 and GST-GhKNL1 proteins. The results suggested that GhKNL1 could be ubiquitinated only in the presence of E1, E2, and GhHUB2, while the absence of any of these components abolished the reaction (Fig. 6A).

To test whether ubiquitination of GhKNL1 by GhHUB2 promoted its degradation, we carried out *in vivo* assays. FLAG tags or FLAG-GhHUB2 were co-infiltrated with MYC-GhKNL1 in tobacco leaves, after which the accumulation of MYC-GhKNL1 was examined by immunoblotting. Compared with co-expression with FLAG tags, co-expression with FLAG-GhHUB2 resulted in less detectable MYC-GhKNL1 (Fig. 6B). Further examination showed that the degradation of GhKNL1 mediated by GhHUB2 could be suppressed by MG132, a 26S proteasome-specific inhibitor (Fig. 6B). This was also confirmed by semi-*in vivo* degradation assays (Fig. 6C). Taken together, the results suggested that GhHUB2 promotes the degradation of GhKNL1 via the ubiquitin-26S proteasome-dependent pathway.

### GhHUB2 regulates the expression of genes involved in fiber development via degradation of GhKNL1

Tissue-specific expression of *GhKNL1* was examined by qRT-PCR and immunoblotting, which showed that *GhKNL1* was predominantly expressed in the cotton fibers (Supplementary Fig. S7A, B). GhKNL1 has been proposed to be a transcriptional repressor (Gong *et al.*, 2014); we tested this assumption using a transient GUS activity assay. GhKNL1 dramatically suppressed the expression of the *GUS* reporter gene, indicating its transcriptional repression activity. This suppression could be abolished by co-expression with GhHUB2, further demonstrating that GhKNL1 is regulated by GhHUB2 (Supplementary Fig. S7C, D).

**Fig. 7.**
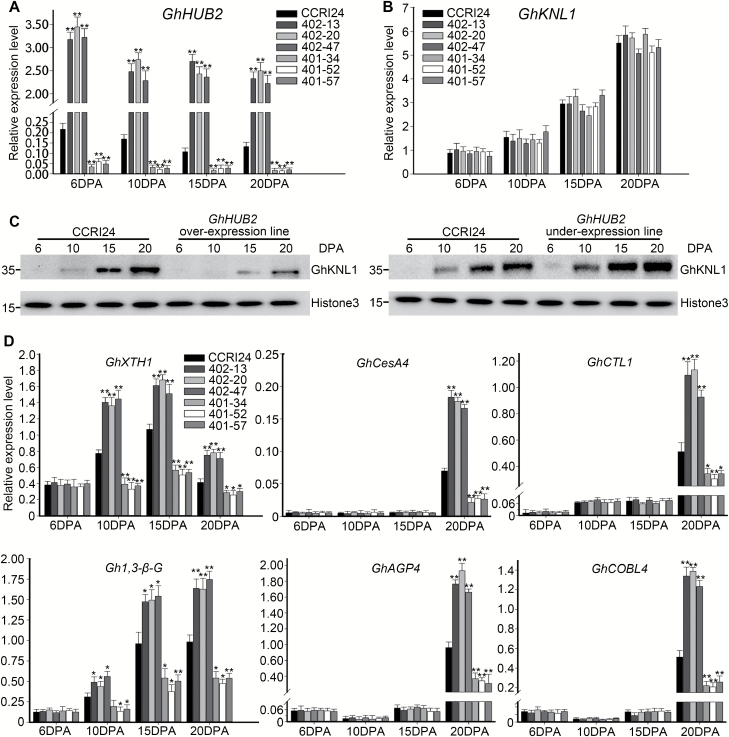
GhHUB2 promotes GhKNL1 degradation and releases GhKNL1-suppressed genes in cotton fibers. (A) qRT-PCR analysis of *GhHUB2* expression in fibers at 6–20 d post-anthesis (DPA). (B) qRT-PCR analysis showing that the RNA transcription level of *GhKNL1* did not change between transgenic lines and the CCRI24 wild-type (WT. (C) GhHUB2 promotes the degradation of GhKNL1 in developing fibers. The abundance of GhKNL1 protein was detected among fiber-extracted nuclear proteins using anti-GhKNL1 antibodies at 6–20 DPA. Cotton Histone3 detected by anti-H3 antibodies was used as a loading control. (D) qRT-PCR analysis of GhKNL1-triggered genes in transgenic cotton fibers. *Histone3* was used as the internal control. Data are means (±SE) of triplicate experiments. Significant differences compared with the WT were determined using Student’s *t*-test; *P<0.05, **P<0.01.

We examined the levels of GhKNL1 in fibers to determine whether the changes in the transgenic lines resulted from its degradation. qRT-PCR showed that the *GhKNL1* transcript levels were comparable between the transgenic lines and the wild-type; they increased from the beginning of the late, rapid-elongation stage at 6 DPA and peaked at the SCW deposition stage at 20 DPA (Fig. 7B). We then investigated the accumulation of GhKNL1 protein in developing fibers and found that, compared with the wild-type, the *GhHUB2*-OE lines always exhibited lower levels throughout fiber development, while the levels in the *GhHUB2*-knockdown lines were elevated (Fig. 7C). The results therefore demonstrated that GhHUB2 controls cotton fiber development by regulating the stability of GhKNL1 via the ubiquitin–26S proteasome pathway.

Overexpression of *GhHUB2* significantly promoted the expression of *GhXTH1* and *Gh1,3-β-G* throughout fiber development (Fig. 7D), which is the opposite effect to that observed when *GhKNL1* is overexpressed (Gong *et al.*, 2014), and knockdown of *GhHUB2* inhibited the expression of *GhXTH1* and *Gh1,3-β-G*, which is the same effect observed with overexpression of *GhKNL1* (Gong *et al.*, 2014). The expression of *GhXTH1* and *Gh1,3-β-G* correlates with fiber length in *GhKNL1* transgenic lines (Gong *et al.*, 2014). We also examined the expression of *GhCesA4*, *GhAGP4*, *GhCTL1*, and *GhCOBL4* in fibers at 6, 10, 15, and 20 DPA and found that these genes were up-regulated in the *GhHUB2*-OE lines but down-regulated in the *GhHUB2*-knockdown lines (Fig. 7D). The expression of *GhCesA4*, *GhAGP4*, *GhCTL1*, and *GhCOBL4* is suppressed in *GhKNL1*-overexpression lines and this has been suggested to contribute to reduced SCW thickness (Gong *et al.*, 2014). Overall, the results suggested that GhHUB2 controls cotton fiber elongation and SCW deposition by degrading GhKNL1 and thus removing GhKNL1-suppression of genes involved in fiber elongation and SCW biosynthesis.

**Fig. 8.**
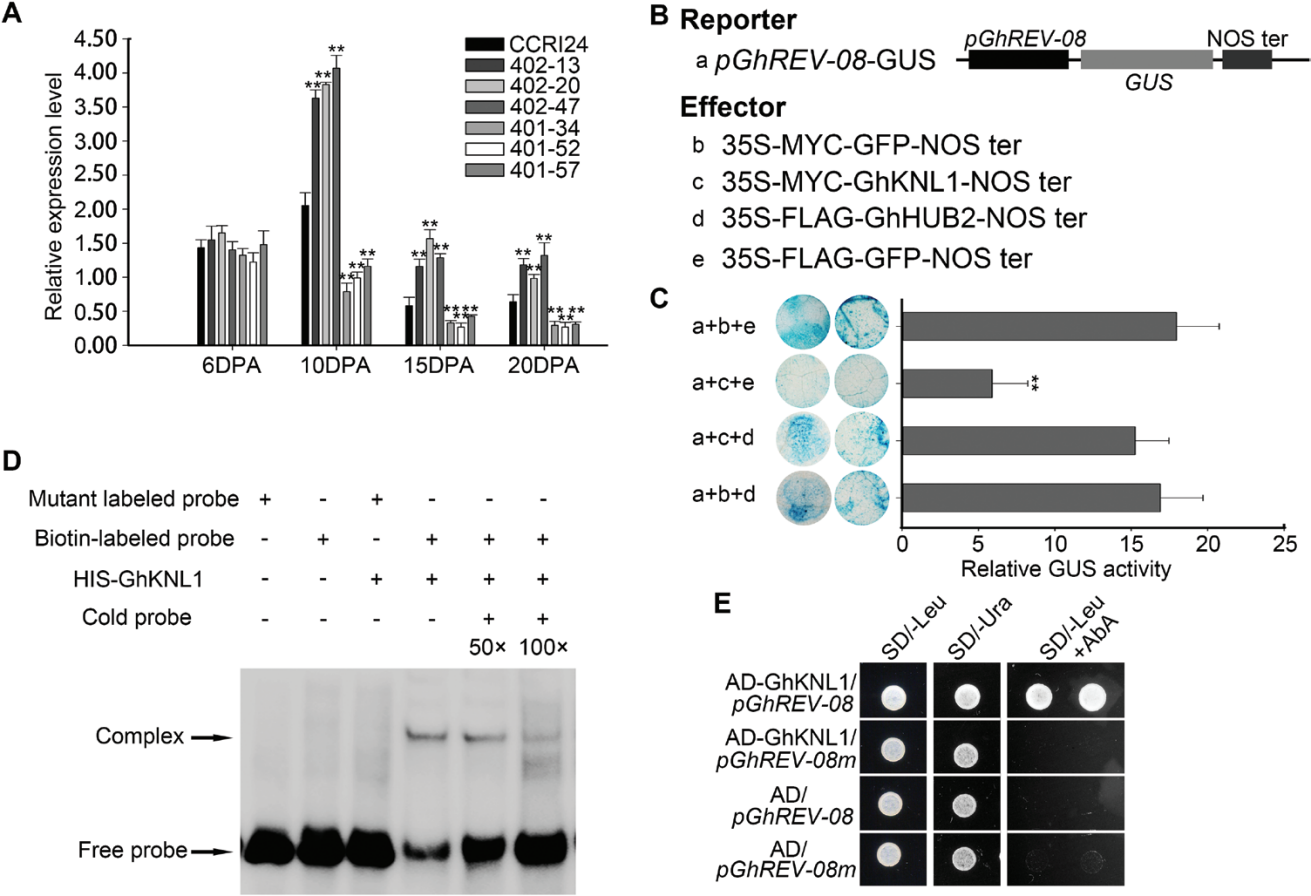
GhHUB2 regulates the expression of *GhREV-08* via GhKNL1. (A) qRT-PCR analysis of *GhREV-08* in wild-type (WT) CCRI24 and transgenic cotton fibers at 6–20 d post-anthesis (DPA). *Histone3* was used as the internal control. Data are means (±SE) of triplicate experiments. Significant differences compared with the WT were determined using Student’s *t*-test; **P<0.01. (B, C) Analysis GhKNL1 repression of the *GhREV-08* promoter by transient GUS activity assays. A 2-kb *GhREV-08* upstream DNA fragment driving *GUS* was used as the reporter (a). The different effectors (b–e) are indicated in (B). Measurement by transient GUS activity assays are shown in (C). Data are means (±SE) of triplicate experiments. Significant differences compared with the WT were determined using Student’s *t*-test; **P<0.01. (D) EMSA results for GhKNL1 binding to a fragment containing the TGAC element from the *GhREV-08* promoter. A mutant labeled probe with a mutation in the TGAC element (TTAC) was used as the negative control. (E) Yeast one-hybrid assay of protein–DNA interactions. *GhKNL1* was cloned into pGADT7 as prey, and a 3× fragment containing a TGAC element from the upstream DNA of *GhREV-08* was cloned into the pAbAi vector (*pGhREV-08*) as bait. The same 3× fragment with a mutation in the TGAC element (TTAC) was also cloned into pAbAi (*pGhREV-08m*). The growth of yeast transformed with the indicated prey and bait on SD/–Leu + AbA medium indicates protein–DNA interactions.

Studies in Arabidopsis have shown that AtKNAT7, a homolog of GhKNL1, binds to the promoter of the homeodomain transcription factor *REVOLUTA* (*AtREV*) and negatively regulates its expression, thus controlling SCW deposition (Liu *et al.*, 2014). We searched the *G. hirsutum* accession TM-1 genome and identified four AtREV homologs (Supplementary Fig. S8A). qRT-PCR suggested that *GhREV-08* was most abundant in developing fibers, with expression being relatively high during fiber initiation and the rapid elongation stage, but dramatically decreasing during the SCW deposition stage. This expression pattern was the opposite to that of *GhKNL1*, indicating that GhKNL1 may negatively regulate the expression of *GhREV-08* during fiber development (Supplementary Fig. S8B). To confirm this possibility, we carried out transient GUS activity assays and found that GhKNL1 significantly repressed the expression of the *GUS* reporter gene driven by the upstream fragment of *GhREV-08* (Fig. 8B, C), demonstrating that GhKNL1 is a transcriptional repressor of *GhREV-08*. Further analysis showed that this repression could be relieved by co-expression with GhHUB2. To determine whether GhKNL1 directly binds to the promoter of *GhREV-08*, we performed EMSAs and found that His-GhKNL1 formed a complex with biotin-labeled fragments from the *GhREV-08* promoter containing the TGAC element (Fig. 8D), which has been reported to be the binding site of KNOX transcription factors (Krusell *et al.*, 1997; Tioni *et al.*, 2005). However, mutation in the TGAC element abolished this interaction. This result was also confirmed by yeast one-hybrid assays (Fig. 8E). These results demonstrated that *GhREV-08* is the direct target of GhKNL1 in cotton fibers. Since GhHUB2 promoted the degradation of GhKNL1, the expression of *GhREV-08* significantly increased in the *GhHUB2*-OE fibers but decreased in the *GhHUB2*-knockdown lines (Fig. 8A).

## Discussion

The developmental process mediated by the ubiquitin–proteasome pathway has been studied in depth in Arabidopsis, but little is known regarding its role in triggering cotton fiber development. In this study, we showed that, in addition to regulating flowering time via H2Bub1, GhHUB2 controls fiber development by triggering the accumulation of GhKNL1. GhHUB2 has no influence on fiber initiation (Supplementary Fig. S9). Overexpression of *GhHUB2* increased fiber length and SCW thickness, while knockdown of *GhHUB2* resulted in shorter fibers and thinner walls (Figs 3, 4). We demonstrated that GhHUB2 interacted with GhKNL1, ubiquitinated GhKNL1, and promoted GhKNL1 degradation to control fiber length and cell wall thickness (Figs 5–7). GhHUB2 up-regulated *GhXTH1* and *Gh1,3-β-G* during the elongation stage, as well as *GhCesA4*, *GhAGP4*, *GhCTL1*, *GhCOBL4*, and *GhREV-08* (Figs 7, 8), the last of which is a direct target of GhKNL1, during the SWC deposition stage (Supplementary Fig. S10). Thus, our study has revealed a novel role of HUB2 in plants and sheds lights on the ubiquitin–proteasome pathway with respect to triggering fiber development.

### A complex role of HUB2 in multiple biological processes

The roles of HUB2 and its homologs BRE1 in yeast and RNF20/40 in humans in acting as chromatin remodeling factors have been studied in depth. Our present study showed that GhHUB2 triggered flowing via the epigenetic modification of H2B in both Arabidopsis and cotton (Fig. 1, Supplementary Fig. S3), demonstrating that, as a homolog of BRE1 and AtHUB2, it plays a conserved role in H2Bub1. In addition, our results also suggested that GhHUB2 directed the ubiquitination of GhKNL1 *in vitro* (Fig. 6A), and using transient protein degradation assays we showed that GhHUB2 promoted the degradation of GhKNL1 *in vivo* (Fig. 6B, C). We also demonstrated that GhHUB2 destabilized GhKNL1 and promoted its degradation in cotton fibers (Fig. 7C). This degradation of GhKNL1 could be inhibited by MG132, a specific 26S proteasome inhibitor, indicating that GhHUB2 plays a role in the ubiquitination–26S proteasome pathway. Indeed, recent studies have also suggested additional involvements with regard to HUB2 and its homologs beyond H2Bub1. Silva *et al.* (2015) reported that BRE1 and its corresponding E2 Rad6 are responsible for K63-linked polyubiquitination of ribosomal proteins, and that this is very important in resistance against oxidative stress. Liu *et al.* (2009) and Ren *et al.* (2014) have suggested that RNF20 promotes the degradation of both the Ebp1 tumor suppressor and the transcription factor AP-2a via polyubiquitination;. Lee *et al.* (2014) have also reported that RNF20 destabilizes SREBP1c via ubiquitination. Thus, our study together with those in yeast and mammalian cells indicates that while HUB2 and its homologs play conserved roles in H2Bub1, they also have other functions that may have diverged throughout evolution.

### GhHUB2 controls fiber development via the ubiquitin–26S proteasome pathway

The development of cotton fibers involves a rapid growth process with active transcription and metabolism. Using transcriptomic and proteomic analyses, previous studies have suggested that the ubiquitin–26S proteosome-dependent pathway may be involved in the regulation of fiber initiation, elongation, and SCW deposition (Yang *et al.*, 2008; Al-Ghazi *et al.*, 2009; Du *et al.*, 2013). Ho *et al.* 2010) and Zhang *et al.* (2003) identified several E2s and RING-type E3s expressed in developing fibers; however, the mechanism that is triggered by these proteins is still unclear. In our present study, we showed that GhHUB2 is a functional E3 ligase that triggers fiber development and is responsible for the ubiquitination and degradation of GhKNL1 via the ubiquitin–26S proteasome-dependent pathway.

The expression of *GhKNL1* reached relatively high levels in fibers by 10–15 DPA (Fig. 7, Supplementary Fig. S7), suggesting that GhKNL1 suppressed the expression of genes involved in promoting fiber elongation during the late elongation stage, thus restricting fiber length. Expression of *GhKNL1* peaked at 20 DPA, the stage of SCW deposition. It has previously been demonstrated that overexpression of *GhKNL1* inhibits the expression of *GhXTH1* and *Gh1,3-β-G*, which are responsible for cell wall loosening and cell expansion, thus reduces cotton fiber length (Gong *et al.*, 2014). Overexpression of *GhKNL1* also suppresses the expression of *GhCesA4*, *GhAGP4*, *GhCTL1*, and *GhCOBL4*, which participate in SCW biosynthesis and deposition, and thus reduces SCW thickness (Gong *et al.*, 2014). Our results showed that GhHUB2 promoted the degradation of GhKNL1 and released its suppression during the late-elongation and SCW deposition stages, resulting in increased fiber length and SCW thickness in *GhHUB2*-OE (overexpression) lines, with opposite results in *GhHUB2*-knockdown lines (Figs 3, 4). The suppressed genes included *GhXTH1*, *Gh1,3-β-G*, *GhCesA4*, *GhAGP4*, *GhCTL1*, and *GhCOBL4*, which were negatively regulated by GhKNL1 (Figs 3, 7D). The genes that participated in biosynthesis of lignin/lignin-like phenolics and SCW formation, such as *Gh4CL1* and *GhCAD6*, whose homologs in Arabidopsis are controlled by AtKNAT7, were also involved (Fig. 4J). We also confirmed that GhKNL1 controlled fiber SCW deposition via modulation of *GhREV-08* expression (Fig. 8). *REV* is the only identified direct target of KNAT7 in Arabidopsis (Liu *et al.*, 2014) and interruption of its expression correlates with irregular SCW deposition (Zhong and Ye, 1999; Ohashi-Ito *et al.*, 2005; Liu *et al.*, 2014). Moreover, *REV* participates in auxin biosynthesis, transport, signal transduction, and responses (Brandt *et al.*, 2012; Reinhart *et al.*, 2013). Changes in the auxin pathway may also contribute to altered fiber qualities in transgenic cotton lines (Zhang *et al.*, 2011*b*). Our results, together with those of previous studies, therefore indicate that a transcription network that involves GhKNL1 triggering SCW deposition exists in cotton fibers and is somewhat similar to the network of AtKNAT7 in Arabidopsis. GhHUB2 may control the SCW transcriptional network via the ubiquitin–26S proteasome pathway by degrading key components such as GhKNL1 in cotton fibers.

While the expression of *GhKNL1* was developmentally regulated (Supplementary Fig. S7A, B), the expression of *GhHUB2* was constitutive, indicating that mechanisms triggering the activity of E3 ligase or stabilizing its substrate may exist to guarantee that GhKNL1 is degraded at the proper time and by the necessary amount. This phenomenon may involve post-translational modifications, including phosphorylation and sumoylation (Miura *et al.*, 2007; Ding *et al.*, 2015; Zhai *et al.*, 2017).

### Possible mechanism involving H2Bub1 in controlling fiber development

We have shown that GhHUB2 participates in the regulation of fiber elongation and SCW deposition, at least partly through the degradation of the transcriptional repressor GhKNL1. We cannot rule out the possibility that GhHUB2 also triggers fiber development via epigenetic modification. In Arabidopsis, H2Bub1 of *FLC* chromatin is essential for its H3K4 and H3K36 methylation, thus stimulating the expression of *FLC* and repressing flowering (Cao *et al.*, 2008; Xu *et al.*, 2009). In rice, H2Bub1 regulates anther development by the enhancement of H3K4me2 in *OsCP1* and *UDT1* chromatin (Cao *et al.*, 2015). These studies demonstrate the essential roles of H2Bub1 and H3 methylation. A recent study has suggested that H3K9me2 mediates DNA methylation in developing cotton fibers and triggers lipid biosynthesis and spatiotemporal production of reactive oxygen species (Wang *et al.*, 2016). Although crosstalk between H2Bub1 and H3K9me2 has not been reported in plants, studies in yeast and human cells indicate that loss of H2Bub1 correlates with increased H3K9me2 and influences gene expression (Sadeghi *et al.*, 2014). In our present study, the expression of *GhRDL1*, *GhKOR1*, *GhPIN1*, and *GhCesA2* significantly changed in transgenic cotton fibers (Figs 3, 4). As these genes have not been reported to be triggered by GhKNL1, whether their altered expressions were due to GhHUB2-triggered epigenetic modification needs further investigation. In addition, recent studies have demonstrated that H2Bub1 triggers stress-induced and defense responses via regulation of microtubule dynamics (Hu *et al.*, 2014; Zhou *et al.*, 2017). The reorganization of microtubules is associated with fiber elongation and SCW deposition (Preuss *et al.*, 2003; Wang *et al.*, 2010), and hence whether H2Bub1 affects fiber development via microtubule reorganization presents an interesting question. The influence of GhHUB2-directed H2Bub1 on fiber development clearly needs to be addressed further.

## Supplementary data

Supplementary data are available at *JXB* online.

Fig. S1. An overview of the *GhHUB2* gene.

Fig. S2. Analysis of tissue-specific expression of *GhHUB2* in transgenic Arabidopsis.

Fig. S3. Analysis flowering time in the transgenic cotton lines.

Fig. S4. GhHUB2 controls fiber development in an *in vitro* ovule culture assay.

Fig. S5. Expression levels of *GhCesA7* and *GhCesA8* in fibers from transgenic lines and the wild-type at 10 DPA and 20 DPA.

Fig. S6. Complete immunoblot blots as shown partially in Fig. 5E.

Fig. S7. Tissue-specific expression and transcriptional repression activity of *GhKNL1*.

Fig. S8. Phylogenetic and tissue-specific expression analysis of *Gossypium hirsutum REVOLUTA* genes (*GhREV*).

Fig. S9. SEM images of the ovule of *GhHUB2* transgenic cotton lines and the wild-type at 0 DPA.

Fig. S10. A proposed model for the regulation of cotton fiber development by GhHUB2 via the degradation of GhKNL1.

Table S1. Comparison of fiber quality parameters between wild-type and transgenic cotton plants.

Table S2. Proteins interacting with GhHUB2 identified by yeast-two hybrid screening.

Table S3. List of antibodies used in this study.

Table S4. Accession numbers relevant to this study.

Table S5. List of oligonucleotide primers used in this study.

Supplementary Tables and Figures
