# Supplementary material for: GhHUB2, a ubiquitin ligase, is involved in cotton fiber development via the ubiquitin–26S proteasome pathway
Source: J Exp Bot. 2018 Jul 20;69(21):5059–75. doi: 10.1093/jxb/ery269 (PMC6184758; doi:10.1093/jxb/ery269)
Supplement: Supplementary Tables and Figures [file ery269_suppl_supplementary_tables-s1-s5_figures-s1-s10.pdf]

Supplementary data

Article title:

GhHUB2, a ubiquitin ligase, is involved in cotton fiber development

Authors: Hao Feng, Xin Li, Hong Chen, Jie Deng, Chaojun Zhang, Ji Liu, Tao Wang, Xueyan Zhang and Jiangli Dong

The following supplementary data is available for this article:

Supplementary tables:

Table S1. Comparison of fiber quality parameters between wild-type and transgenic cotton plants

Samples	Fiber length (mm)	Fiber strength (cN/tex)	Length uniformity (%)	Micronaire value
CCRI24	29.44±0.49	31.73±0.82	85.47±0.96	4.24±0.18
402-13	31.69±0.62**	33.79±0.87**	85.37±1.01	4.92±0.21**
402-20	31.97±0.74**	33.92±0.91**	85.31±1.15	4.97±0.23**
402-47	31.62±0.51**	33.35±0.74*	85.41±1.06	4.83±0.16**
401-34	27.75±0.36**	29.37±0.76**	86.14±0.83	3.46±0.16**
401-52	27.37±0.48**	29.14±0.88**	85.48±0.94	3.35±0.17**
401-57	27.63±0.44**	29.56±0.81*	85.51±1.07	3.60±0.17**

Values are mean ±SD for samples of wild-type (CCRI24) and *GhHUB2* overexpression plants (lines 402-13, 402-20, and 402-47) and underexpression plants (lines 401-34, 401-52 and 401-57). The fiber length, fiber strength, length uniformity and micronaire value were measured at the Center of Cotton Fiber Quality Inspection and Testing, Chinese Ministry of Agriculture (Anyang, Henan province, China). **$P < 0.01$, *$P < 0.05$, Student's *t*-test. cN/tex: Centi-Newton per Tex.

Table S2. Proteins interacting with GhHUB2 identified by yeast-two hybrid screen

Putative protein type	Average frequency
E3 ubiquitin-protein ligase BRE1-like 1 (HUB1)	8
Homeobox protein knotted-1-like 7 (GhKNL1)	7
Ubiquitin conjugating enzyme 1	6
Cell division protein ftsH	6
SINA protein	6
Histone H2B	4
Elongation factor 1-alpha	4
Chromatin remodeling 24	3
Serine/threonine-protein kinase SRK2B	3
DNA damage repair/toleration protein	3
P-loop containing nucleoside triphosphate hydrolases superfamily	3
Splicing factor isoform	3
Phosphatase 2C family	3
GTP-binding nuclear protein Ran-3	3
Glycine-rich RNA-binding protein 3	2
AP2/ERF domain-containing transcription factor	2
Nucleolin like 2 isoform	2
RING/FYVE/PHD zinc finger superfamily protein	2
Phragmoplast-associated kinesin-related protein	2

Yeast-two hybrid assay was repeated using GhHUB2. Average frequency indicates the average numbers of clones obtained for each protein.

Table S3. List of antibodies used in this investigation

Antibody	Product code	Dilutions
anti-FLAG	F3165, Sigma-Aldrich	1:10000
anti-HA	CB100005M, Cali-Bio	1:2000
anti-MYC	M4439, Sigma-Aldrich	1:10000
anti-H3	BE3015, EasyBio	1:5000
anti-ACTIN	0125, CW-Bio	1:5000
anti-H2Bub1	5546S, CST	1:1000
anti-His	66005-1-Ig, Proteintech	1:10000
anti-GST	66001-1-Ig, Proteintech	1:10000
anti-Ubi	ab7254, Abcam	1:5000
Peroxidase-labeled goat anti-mouse IgG (H + L) secondary antibodies	5220-0341, KPL	1:5000
Peroxidase-labeled goat anti-rabbit IgG (H + L) secondary antibodies	074-1506, KPL	1:5000

Table S4. Accession numbers

Gene or protein	Accession number
AtACT2	AT3G18780
AtHUB2	AT1G55250
MAF10	AT1G7708
MAF2	AT5G65050
MAF3	AT5G65060
MAF4	AT5G65070
MAF5	AT5G65080
AtSOC1	AT2G45660
AtFT	AT1G65480
NbEF1α	AY206004
GhHOX3	KJ595847
GhSINA	NM_001327228
GhFT	HM631972
GhSOC1	NM_001327654
GhSOC1-like	XM_016831064
GhRDL1	AY072821
GhXTH1	HM749062
GhKOR1	AY574906
GhPIN1	KJ126841
GhEXPA1	AF043284
GhCesA1	U58283
GhCesA2	AAF72619
GhCesA3	AF150630
GhCesA4	U58284
GhCesA6	JQ345694
GhCesA7	Gh_A05G3965
GhCesA8	Gh_A10G0327
GhPAL1	NM_001326949
Gh4CL1	FJ479707
GhCCR1	FJ376603
GhCAD6	NM_119958
GhHIS3	AF024716
GhCOBL4	CO496020
GhCTL1	AY291285
GhAGP4	EF470295
Gh1,3-β-G	CAA92278
GhREV-03	XM_016898222
GhREV-05	XM_016894979
GhREV-08	XM_016899287
GhREV-13	XM_016868724
AtHUB1	AT2G44950
AtHUB2	AT1G55250
OsHUB1	ADS97227
OsHUB2	A2ZAC2

Table S5. List of oligonucleotide primers used in this investigation

Primer	Sequence(5'-3')	Purpose
GhHUB2-F	ATGGAAAGCTTTGAATCTGAAGAAC	Gene cloning
GhHUB2-R	TCATATTTTCACAAACCGAACATC	
GhKNL1-F	ATGCAAGAACCAGGGTTAGGTATG	Gene cloning
GhKNL1-R	CTACCGTTTTCGCTTCGACTTTAAAG	
GhHUB2-KpnI-F	GGTACCATGGATTACAAAGATCATGATGGTGACTATAAGGACCAC GACATCGATTACAAAGATGATGATGATAAAATGGAAAGCTTTGA ATCTGAAG	Cotton transformation
GhHUB2-SalI-R	GTCGACTCATATTTTCACAAACCGAACATCA	
GhHUB2-RNAi-F	ACTGCAATACAGAAGCTTCAGGATGA	Cotton transformation
GhHUB2-RNAi-R	CATATTTTCACAAACCGAACATC	
402-F	ATGGAAAGCTTTGAATCTGAAGAAC	Transgene identification
402-R	TCATATTTTCACAAACCGAACATCA	
401-F	GATGCAGTCAAAAGATTCAGGAC	Transgene identification
401-R	CATATTTTCACAAACCGAACATCATT	
GhHUB2-EcoRI-F	GAATTCATGGAAAGCTTTGAATCTGAAGAAC	Protein expression
GhHUB2-SalI-R	GTCGACTCATATTTTCACAAACCGAACATCA	
GhHUB2-BamHI-F	GGATCCATGGAAAGCTTTGAATCTGAAGAAC	Subcellular localization
GhHUB2-SalI-R	GTCGACTCATATTTTCACAAACCGAACATCA	
GhHUB2-DONR-F	GGGGACAAGTTTGTACAAAAAAGCAGGCTTGATGGAAAGCTTT GAATCTGAAGAAC	Yeast-two hybrid
GhHUB2-DONR-R	GGGGACCACTTTGTACAAGAAAGCTGGGTTCATATTTTCACAAA CCGAA CATC	
GhHUB2-EcoRI-F	GAATTCATGGAAAGCTTTGAATCTGAAGAAC	Yeast-two hybrid
GhHUB2-BamHI-R	GGATCCTCATATTTTCACAAACCGAACATCA	
GhHUB2-BamHI-F	GGATCCATGGAAAGCTTTGAATCTGAAGAAC	Firefly luciferase complementation
GhHUB2-KpnI-R	GGTACCTCATATTTTCACAAACCGAACATCA	
GhHOX3-KpnI-F	GGTACCATGGATTGCGGAAGCGGCGGCGGC	Firefly luciferase complementation
GhHOX3-BglII-R	AGATCTGAGAACTAGGACAATTCAAAGCAG	
GhSINA-BamHI-F	GGATCCATGGAGTCGGATACTATCGAGTGC	Firefly luciferase complementation
GhSINA-KpnI-R	GGTACCGTTTGGTATGCACATGTTGGCATC	
GhKNL1-KpnI-F	GGTACCATGCAAGAACCAGGGTTAGCTAT	Firefly luciferase complementation
GhKNL1-SalI-R	GTCGACCCGTTTTCGCTTCGACTTTAAAG	
GhKNL1-EcoRI-F	GAATTCATGCAAGAACCAGGGTTAGCTAT	Subcellular localization
GhKNL1-KpnI-R	GGTACCGCCGTTTTCGCTTCGACTTTAAAG	
GhKNL1-EcoRI-F	GAATTCATGCAAGAACCAGGGTTAGCTAT	Yeast-two hybrid
GhKNL1-SalI-R	GTCGACCTACCGTTTTCGCTTCGACTTTAAAG	
GhKNL1-EcoRI-F	GAATTCATGCAAGAACCAGGGTTAGCTAT	Yeast-two hybrid
GhKNL1-BamHI-R	GGATCCCTACCGTTTTCGCTTCGACTTTAAAG	
GhKNL1-BamHI-F	GGATCCATGCAAGAACCAGGGTTAGCTAT	Protein expression
GhKNL1-SalI-R	GTCGACCTACCGTTTTCGCTTCGACTTTAAAG	
GhKNL1-KpnI-F	GGTACCCATGCAAGAACCAGGGTTAGCTAT	Transient expression
GhKNL1-BamHI-R	GGATCCCTACCGTTTTCGCTTCGACTTTA	
pGhHUB2-SalI-F	GTCGACGCAGTACCATACAACTGCTCCAGCTC	Tissue-specific expression
PGhHUB2-HindIII-R	AAGCTTACTGCAGTTAGGATTGCGATTTAC	
pGhREV-08-BamHI-F	GGATCCAGCATACCAATAATAAGTCACACAAGC	Transient expression
pGhREV-08-SalI-R	GTCGACCCATTTCTTGCTAAAACTCAATCTTTC	

pGhREV-08-ABAi-F	TCACTAGCAGCTTGACTGATGATCATCATCTCACTAGCAGCTTGA CTGATGATCATCATCTCACTAGCAGCTTGACTGATGATCATCATC	Yeast-one hybrid
pGhREV-08-ABAi-R	GATGATGATCATCAGTCAAGCTGCTAGTGAGATGATGATCATCAG TCAAGCTGCTAGTGAGATGATGATCATCAGTCAAGCTGCTAGTG AAGCT	
Mutant pGhREV-08-ABAi-F	TCACTAGCAGCTTTACTGATGATCATCATCTCACTAGCAGCTTTAC TGATGATCATCATCTCACTAGCAGCTTTACTGATGATCATCATC	Yeast-one hybrid
Mutant pGhREV-08-ABAi-R	GATGATGATCATCAGTAAAGCTGCTAGTGAGATGATGATCATCAG TAAAGCTGCTAGTGAGATGATGATCATCAGTAAAGCTGCTAGTGA AGCT	
pGhREV-08-probe-F	TGTTTCTAGCTGATGATGATCATCAGTCAAGCTGCTAGTGAAATG AAATGAAATGAAA	EMSA
pGhREV-08-probe-R	TTTCATTTCATTTCATTTCACTAGCAGCTTGACTGATGATCATCATC AGCTAGAAACA	
Mutant pGhREV-08-probe-F	TGTTTCTAGCTGATGATGATCATCAGTAAAGCTGCTAGTGAAATG AAATGAAATGAAA	EMSA
Mutant pGhREV-08-probe-R	TTTCATTTCATTTCATTTCACTAGCAGCTTTACTGATGATCATCATC AGCTAGAAACA	
GhFT-RT-F	TCTGCTATGAGAGCCCACGA	qPCR
GhFT-RT-R	TCATGTCCTACGGCCACCGGATCCACT	
GhSOC1-RT-F	AGCATGCAGTGGCAGCATCTGA	qPCR
GhSOC1-RT-R	TGGCTCTGACGCGGGTTACG	
GhSOC1-like-RT-F	CTCAAATTGAAAGATACACACAG	qPCR
GhSOC1-like-RT-R	GTTCTGCTGGCTGCATGTTGTAC	
GhHUB2-RT-F	GATGAGTTCCAAATGAAGTTGG	qPCR
GhHUB2-RT-R	GTCAAAACACACACCACATTTGAG	
GhKNL1-RT-F	GCCAAACTTGTGGAGGAAACAG	qPCR
GhKNL1-RT-R	GAGACATACATCACAATAATGAG	
NbEF1α-RT-F	ACTGCACTGTGATTGATGCC	RT-PCR
NbEF1α-RT-R	GACACCAGTTTCCACACGAC	
GhHIS3-RT-F	GGCATACCTTGTGGGTCTTTTTGA	qPCR
GhHIS3-RT-R	CTACCACTACCATCATGGC	
GhRDL1-RT-F	ATACCGTTTTCATCTGACAAGTTGC	qPCR
GhRDL1-RT-R	CAGCTGCTATTGTATACTTTTGCA	
GhEXPA1-RT-F	GCAGGACTATCACAGCCTACAA	qPCR
GhEXPA1-RT-R	ATGGCACTTGCTCGCCTATTT	
GhHOX3-RT-F	ATTATGAATGGCTTAGCTTTGG	qPCR
GhHOX3-RT-R	ACTGCGTTGCCGTTGGATAG	
GhPAL-RT-F	TCGGTGGTGAGACCTTAACC	qPCR
GhPAL-RT-R	ATCAAGGACCCAATCAGCAC	
GhXTH1-RT-F	GTGCCAGGCCCAGCAA	qPCR
GhXTH1-RT-R	GGCATTAAGGGCTTGATAAGTAGATC	
Gh1,3- ß-G-RT-F	GGTTTGAACGGCAACAATCT	qPCR
Gh1,3- ß-G-RT-R	TTGATCTTTTGCGAGGCTTT	
GhAGP4-RT-F	ACCGGGAATGTCTAGGAAGG	qPCR
GhAGP4-RT-R	CCATAGGCAATAAGTGGAGG	
GhCesA1-RT-F	TGGACTACCCGGTGGATAAGGT	qPCR
GhCesA1-RT-R	CTTTCTTGCAAAGTCGGCTGTT	
GhCesA2-RT-F	TCTGATAATACTGAACATGGTCGGAGT	qPCR
GhCesA2-RT-R	GAAATTAAATTGAACCAACAAAATCATAGG	

GhCesA3-RT-F	ACAGTTTTGGGTTATCGGTGG	qPCR
GhCesA3-RT-R	ATGATGAGGAGAGTGGTTGGTG	
GhCesA4-RT-F	GCAGCAGACGATACAGAATTCG	qPCR
GhCesA4-RT-R	CGTTGTTGATTGCGTCTGAAAC	
GhCesA6-RT-F	TTCTCATTCCTCCAACCACAGT	qPCR
GhCesA6-RT-R	TTGACGACCCACCAAACCT	
GhCesA7-RT-F	CTAGCTGGTATTGACACTAATTT	qPCR
GhCesA7-RT-R	GAGAAGATTGAAGCCAATAGCA	
GhCesA8-RT-F	CCGGATTCTCTGATGCCCTC	qPCR
GhCesA8-RT-R	CAGAAGCCAACAACACTGACC	
Gh4CL1-RT-F	TTGTTGGCTAAGAATCCTGT	qPCR
Gh4CL1-RT-R	GCATTCCTAACCACTGTCCC	
GhCCR1-RT-F	CAAGAAAAGCAGGTCCAGCC	qPCR
GhCCR1-RT-R	CCTACAACACCAAATAAGAT	
GhCAD6-RT-F	CCCTCTTCAGTTTGTTTCCC	qPCR
GhCAD6-RT-R	TCGTTCTTCTCCAGCCTCTC	
GhCTL1-RT-F	CCGACCAAGAACGACACGTT	qPCR
GhCTL1-RT-R	ACCTCGCCCACAAACTTGAT	
GhCOBL4-RT-F	GGCATGAAGTTCTACAATGAC	qPCR
GhCOBL4-RT-R	AATCACCAAACAGTGACCAAC	
GhKOR1-RT-F	TGTTTCCTACACCACCACCA	qPCR
GhKOR1-RT-R	CCACGAGTTTTGTCCAGGTT	
GhPIN1-RT-F	GGACTCCGTGGCGTTCTCTTAC	qPCR
GhPIN1-RT-R	AGACAAGCGTTATGGGCAAGGC	
GhREV-03-RT-F	CAGATATGCAGTGGAATCGATGAG	qPCR
GhREV-03-RT-R	CACCAAGTCTCTCAGACATACAATC	
GhREV-05-RT-F	CATATTTACAGCTGTATGTAATGTTC	qPCR
GhREV-05-RT-R	AACACGAAATCCAGAAGGTAGTAG	
GhREV-08-RT-F	CATTATCATCTTCGCAGCTATGCAG	qPCR
GhREV-08-RT-R	GTGAAGTGCGGTGAAACCTAACTG	
GhREV-13-RT-F	GAAGCTTCAAGGCTTCAAACAGT	qPCR
GhREV-13-RT-R	CATCTGGACCCAATTGACAGCAG	
AtACT2-RT-F	CTCTCCTTGTACGCCAGTGGTC	qPCR
AtACT2-RT-R	TAAGGTCACGTCCAGCAAGGTC	
AtMAF1-RT-F	TCACCTTAAACTCAAAGCCTGATTC	qPCR
AtMAF1-RT-R	CAAACTCTGATCTTGTCTCCGAAG	
AtMAF2-RT-F	CATTGTGGGTCTCCGGTGATTAG	qPCR
AtMAF2-RT-R	GATGAGACCATTGCGTCGTTTG	
AtMAF3-RT-F	TATCTTCCTCGCGCCAATG	qPCR
AtMAF3-RT-R	AGCACAAGAACTCTGATATTTGTCTAC	
AtMAF4-RT-F	GCTTCTCAAGTAACCACCATCAC	qPCR
AtMAF4-RT-R	CTTGGATGACTTTTCCGTAGCAG	
AtMA5-RT-F	CATGGATTGTGCTAGAAAACAACTG	qPCR
AtMAF5-RT-R	GCTTCACTCTTCCGACACATCTAATC	
hub2-2-R1	ATGCTAACAAAGGCAGACGAACAG	RT-PCR
hub2-2-R2	TTCGAGGCTGATAACGAGGTGACG	
AtFT-RT-F	CTTGGCAGGCAAACAGTGTATGCAC	qPCR
AtFT-RT-R	GCCACTCTCCCTCTGACAATTGTAGA	
AtSOC1-RT-F	AGCTGCAGAAAACGAGAAGCTCTCTG	qPCR
AtSOC1-RT-R	GGGCTACTCTCTTCATCACCTCTTCC	

Supplementary figures:

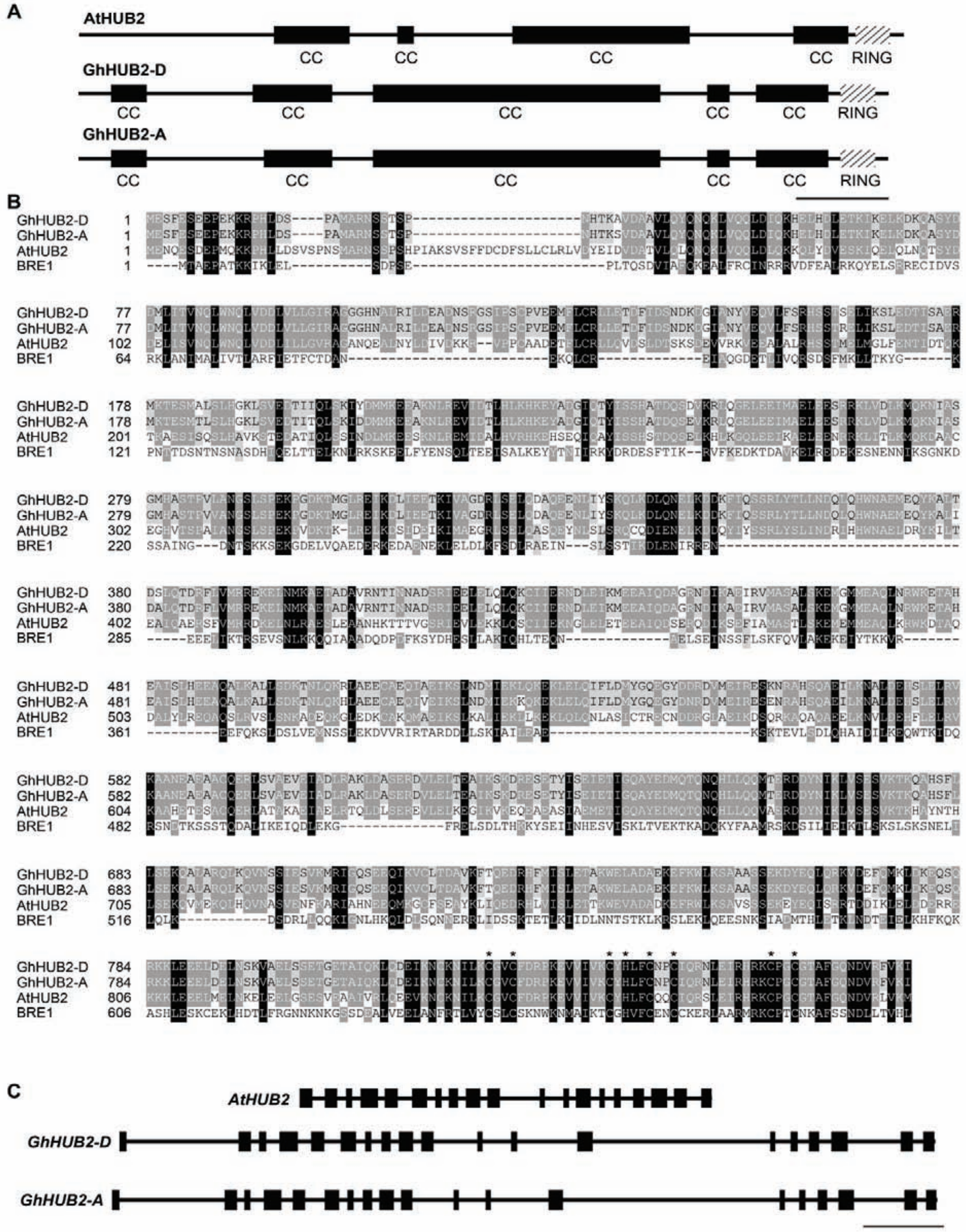

Fig. S1. Overview of *GhHUB2* gene. **(A)** Conserved domains of GhHUB2-A, GhHUB2-D and

AtHUB2. All proteins contain a C3HC4 RING finger domain (RING) to its C terminal. GhHUB2 contain five coiled coil domains (CC) while AtHUB2 contains four coiled coil domains. Scale bar, 100 aa. **(B)** Alignment of amino acid sequences of GhHUB2-A (GenBank: AF530913), GhHUB2-D (GenBank: AF530914), *Arabidopsis thaliana* AtHUB2 (GenBank: L32873) and yeast BRE1 (GenBank: Q07457). All proteins aligned here belong to the C3HC4 RING finger family. The homoeologous GhHUB2-A and GhHUB2-D are 98.4% identical with amino acid sequences. Protein sequences were aligned by CLUSTALW following the default parameters. Conserved amino acids are indicated. **(C)** Schematic show of cotton (*Gossypium hirsutum*) *HUB2* gene, *GhHUB2* and *AtHUB2*. The length of both copies of *GhHHUB2* coding region is around 10 kb, containing 19 exons and 18 introns. Scale bar, 1000bp.

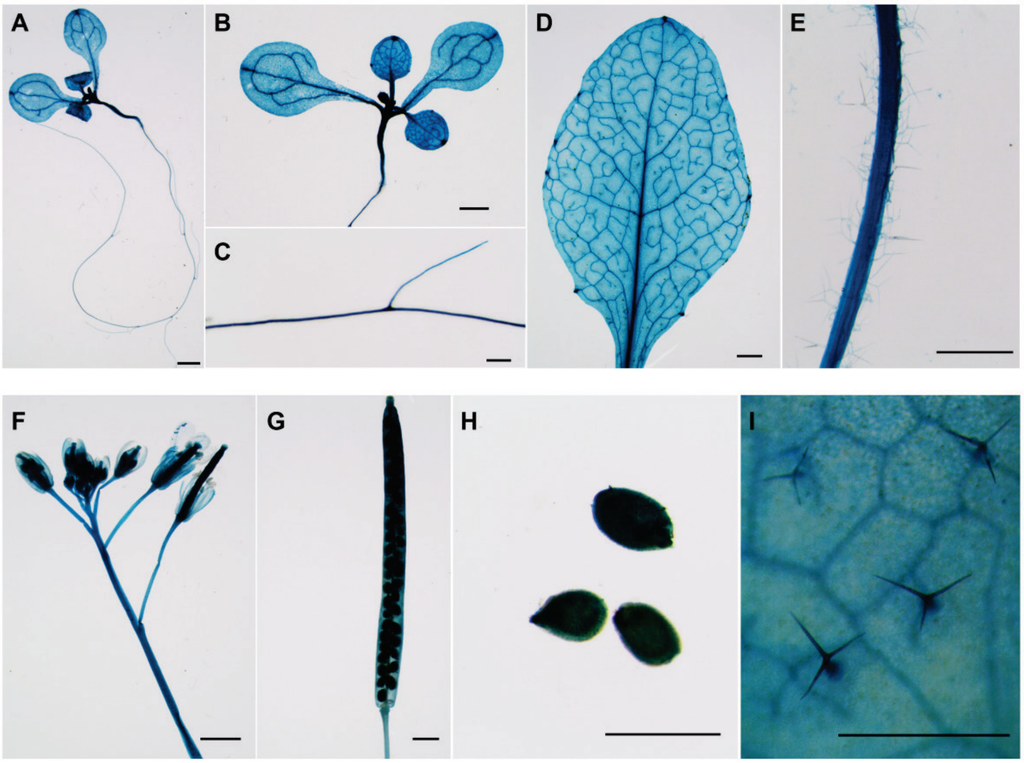

Fig. S2. Analysis of tissue-specific expression of *GhHUB2* in transgenic *Arabidopsis*. GUS staining of *Arabidopsis* seedling **(A)**, cotyledon **(B)**, root **(C)**, leaf **(D)**, stem **(E)**, flower **(F)**, silique **(G)**, seeds **(H)** and trichome **(I)** are shown. Scale bar, 1 mm.

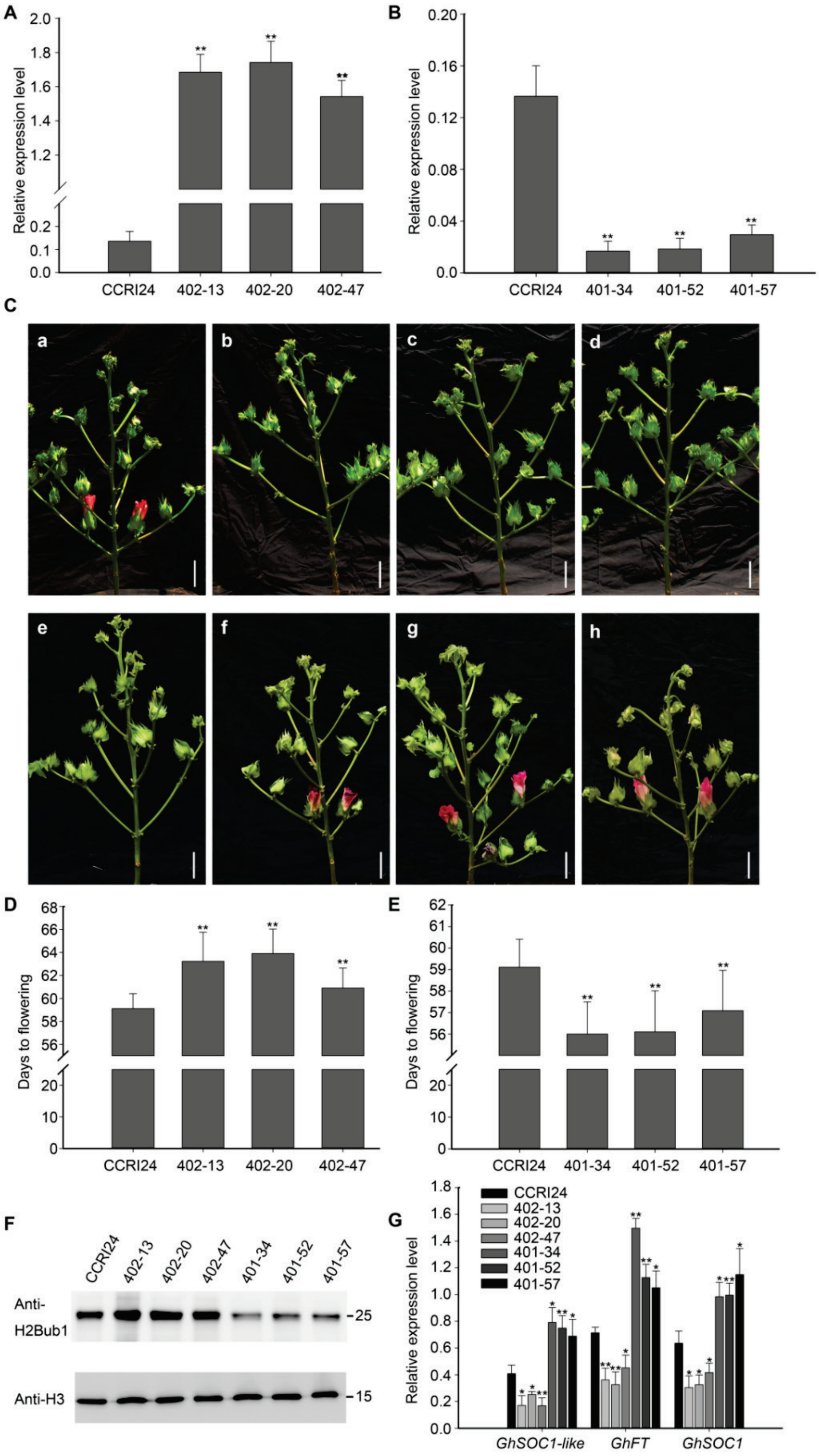

Fig. S3. Analysis flowering time of transgenic cotton. **(A)** Expression of *GhHUB2* in wild-type and *GhHUB2* overexpression cotton leaves. **(B)** Expression of *GhHUB2* in wild-type and

GhHUB2 underexpression cotton leaves. *Histone3* was used as an internal control. **(C)** Phenotype of transgenic lines and wild-type at flowering. (a)-(d) showing delayed flowering time in *GhHUB2* overexpression cotton lines 402-13 (b), 402-20 (c) and 402-47 (d) compared to wild-type (a); (e)-(h) showing earlier flowering time in *GhHUB2* underexpression cotton lines 401-34 (f), 401-52 (g) and 401-57 (h) compared to wild-type (e). Bar = 5cm. **(D)** and **(E)** Measurement of flowering time in transgenic cotton and wild-type. Flowering time was recorded as days from germination until flowering. Error bars indicate the SDs, n≥30 (** $P < 0.01$, Student's *t*-test). **(F)** Analysis the level of H2Bub1 in transgenic cotton and wild-type by immunoblotting with nuclear protein extracted from leaves. Cotton Histone3 was used as a loading control. **(G)** Analysis the expression of *GhFT*, *GhSOC1* and *GhSOC1-like* in transgenic cotton and wild-type. *Histone3* was used as an internal control. Error bars indicate the SEs of triplicate experiments. (** $P < 0.01$, * $P < 0.05$, Student's *t*-test).

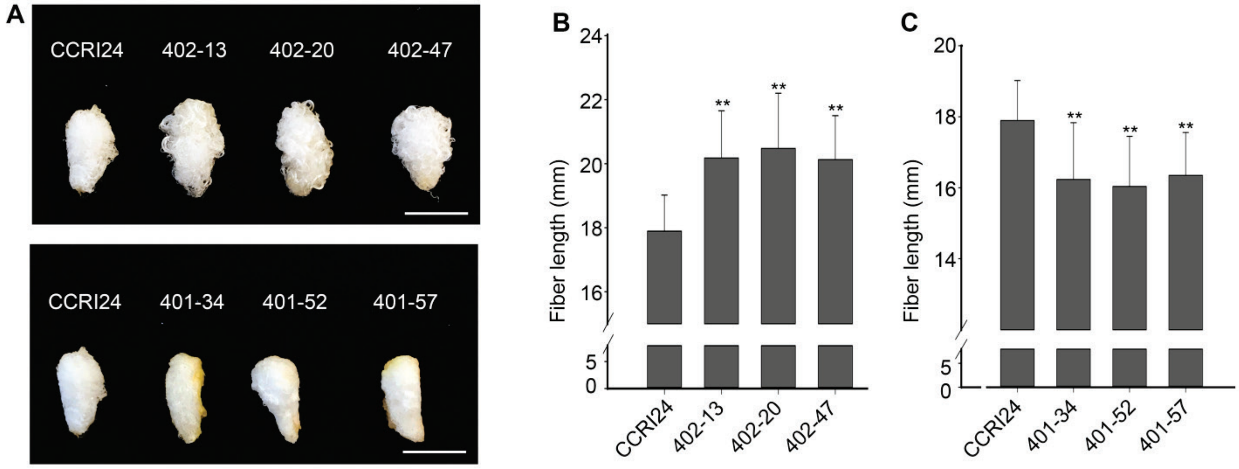

Fig. S4. GhHUB2 controls fiber development in an *in vitro* ovule culture assay. **(A)** Phenotypes of transgenic and wild-type cotton ovules cultured for 20 d in BT medium. Scale bar, 10 mm. **(B)** and **(C)** Fiber lengths of *GhHUB2* overexpression ovules **(B)** and *GhHUB2* underexpression ovules **(C)** cultured for 20 d. Error bars indicate the SEs of triplicate experiments. For each measurement, n≥30 (** $P < 0.01$, Student's *t*-test).

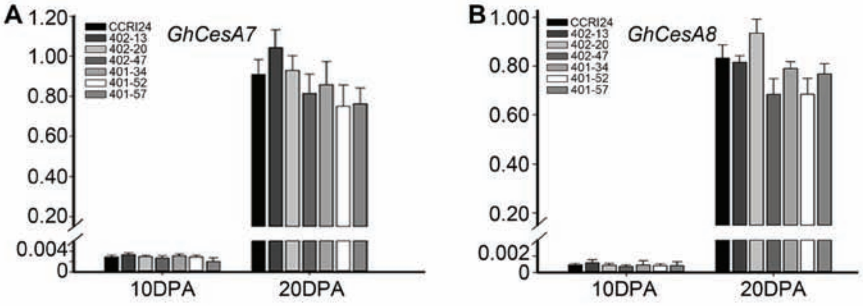

Fig. S5. Expression level of *GhCesA7* and *GhCesA8* involved in fibers from transgenic lines and wild-type at 10 DPA and 20 DPA. *Histone3* was used as an internal control. Error bars indicate the SEs of triplicate experiments.

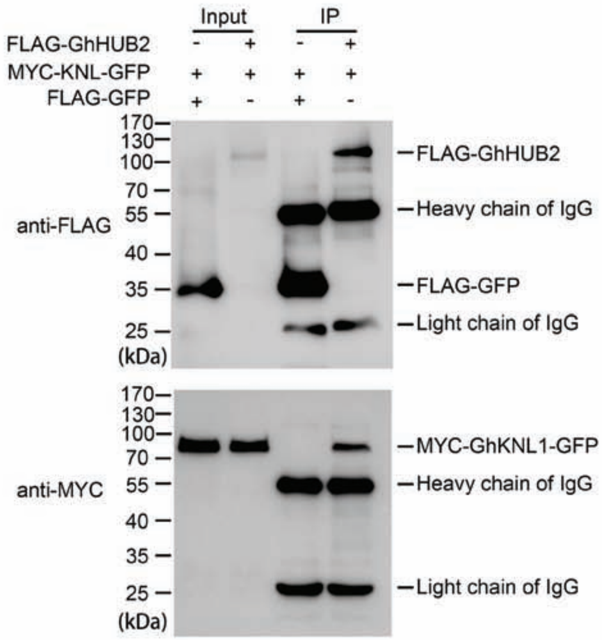

Fig. S6. The whole view of immunoblot blots partially shown in Fig. 5E. Bands at 55 kDa and 25 kDa after immunoprecipitation (IP) are heavy chain and light chain of IgG, respectively.

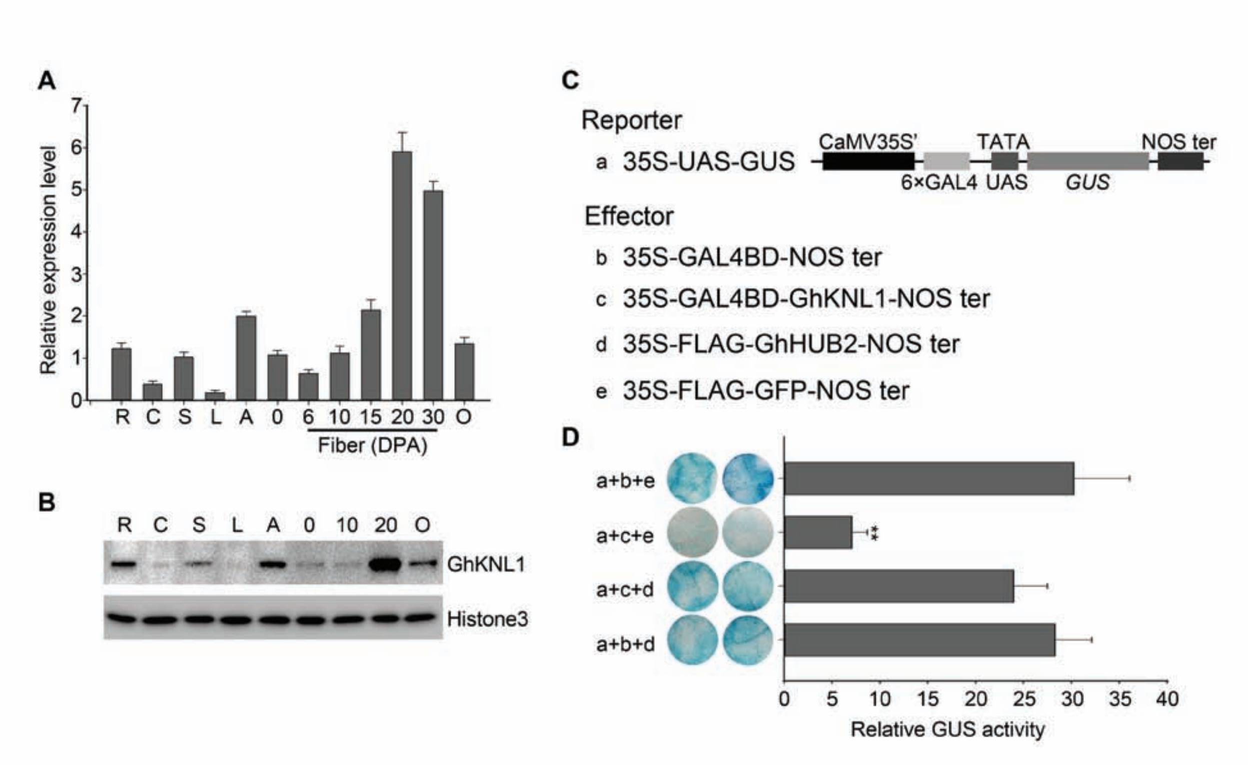

Fig. S7. Tissue-specific expression and transcriptional repression activity of *GhKNL1*. **(A)** qRT-PCR analysis of tissue specific expression of *GhKNL1*. The expression of *GhKNL1* in root (R), cotyledon (C), stem (S), leaf (L), anther (A), 0 DPA ovule (0), 20 DPA ovule (O), and fibers at 6, 10, 15, 20, and 30 DPA are examined. *Histone3* was used as an internal control. Error bars indicate the SEs of triplicate experiments. **(B)** Analysis tissue-specific expression of GhKNL1 by immunoblotting using anti-GhKNL1 antibody. GhKNL1 protein was detected in nuclear protein from different tissues. Cotton Histne3 was used as a loading control. **(C)** and **(D)** Analysis the transcriptional repression activity of GhKNL1 by transient GUS activity assay. GAL4BD-GhKNL1 or GAL4BD used as effector was co-expressed with the reporter 35S-UAS-GUS. Other effectors are shown in **(C)**. GUS activities measured from each combination are shown in **(D)**. Error bars indicate the SEs of triplicate experiments. (**P < 0.01, Student's *t*-test).

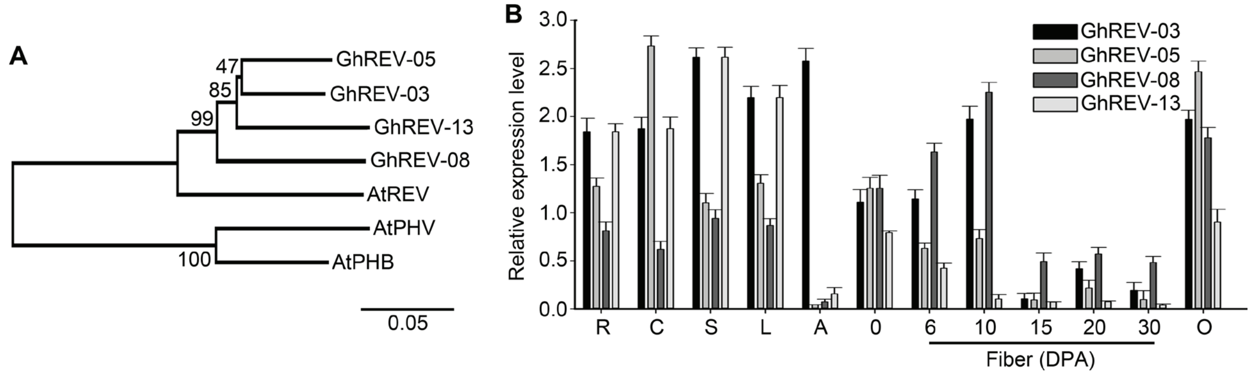

Fig. S8. Phylogenetic and tissue-specific expression analysis of *Gossypium hirsutum REVOLUTA* genes (*GhREV*). **(A)** Phylogenetic tree showing the relationships between cotton GhREV and *Arabidopsis* AtREV. **(B)** Analysis tissue-specific expression of *GhREV* homologs in root (R), cotyledon (C), stem (S), leaf (L), anther (A), 0 DPA ovule (0), 20 DPA ovule (O), and fibers at 6, 10, 15, 20, and 30 DPA by qRT-PCR. *Histone3* was used as an internal control. Error bars indicate the SEs of triplicate experiments.

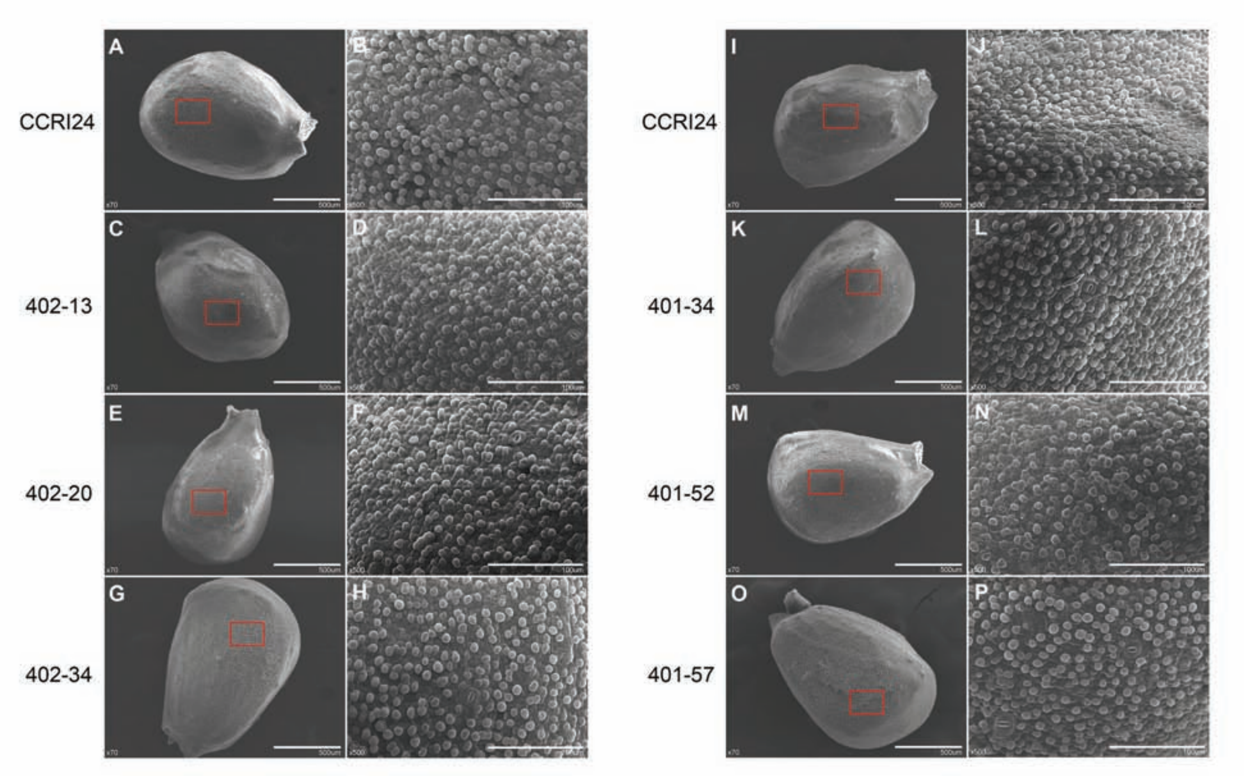

Fig. S9. Scanning electron microscope (SEM) images of the ovule (0 DPA) of *GhHUB2* transgenic cotton lines and the wild-type. B, D, F, H, J, L, N and P (Bar = 100 μm) are magnified frames in A, C, E, G, I, K, M and O (Bar = 500 μm), respectively.

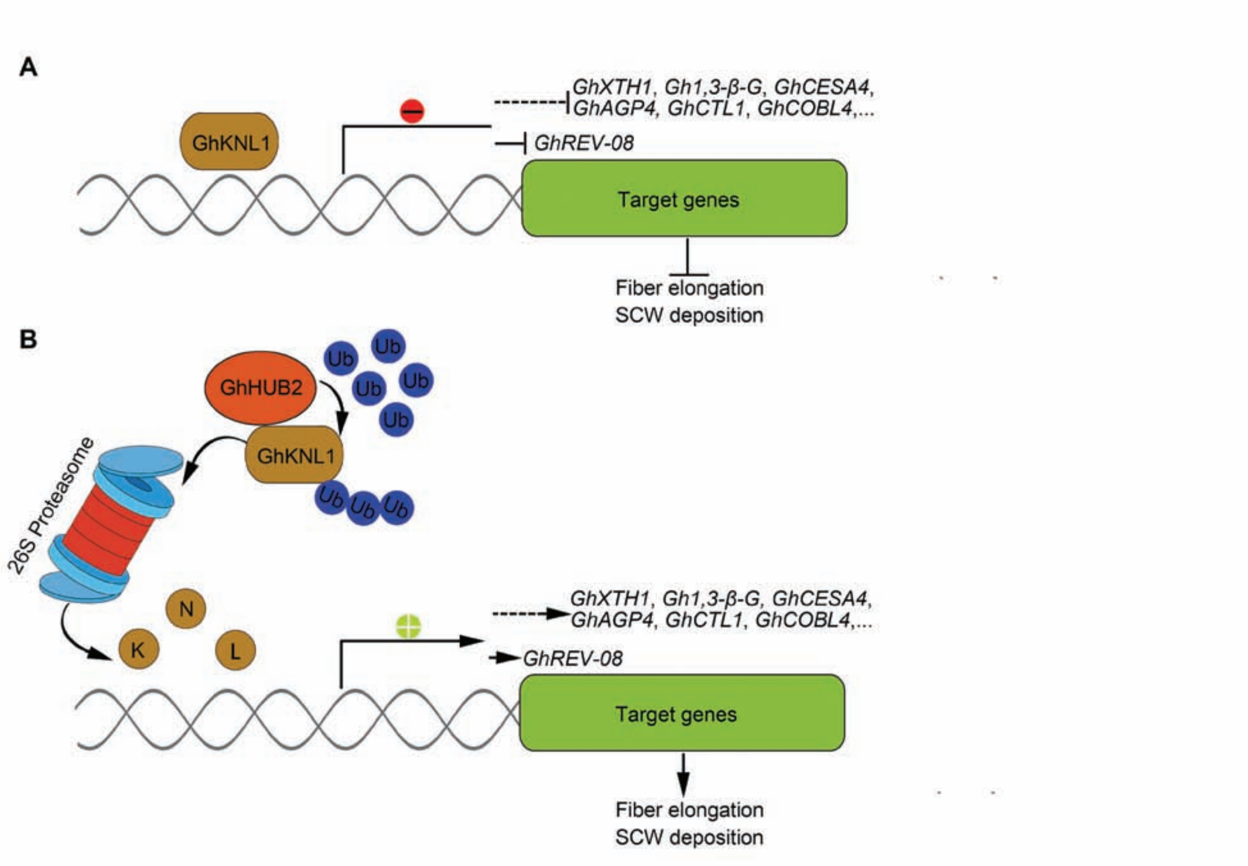

Fig. S10. A proposed model for the regulation of cotton fiber development by GhHUB2 via the degradation of GhKNL1. **(A)** During cotton fiber development, GhKNL1 suppresses the expression of genes involed in fiber elongation and SCW deposition, including *GhXTH1*, *Gh1,3-β-G*, *GhCesA4*, *GhAGP4*, *GhCTL1*, *GhCOBL4* and its direct target *GhREV-08*. This inhibition results in a restricted fiber length and SWC thickness. **(B)** GhHUB2 ubiquitinates and degrades GhKNL1 through the ubiquitin-26S proteasome pathway. The degradation of GhKNL1 promotes fiber elongation and SCW deposition by disinhibition target genes expression. A native balancing mechanism that fine tunes GhHUB2 and GhKNL1 may exist to guarantee proper fiber length and SCW thickness.
